# The uterine and vascular actions of estetrol delineate a distinctive profile of estrogen receptor α modulation, uncoupling nuclear and membrane activation

**DOI:** 10.15252/emmm.201404112

**Published:** 2014-09-11

**Authors:** Anne Abot, Coralie Fontaine, Mélissa Buscato, Romain Solinhac, Gilles Flouriot, Aurélie Fabre, Anne Drougard, Shyamala Rajan, Muriel Laine, Alain Milon, Isabelle Muller, Daniel Henrion, Marine Adlanmerini, Marie-Cécile Valéra, Anne Gompel, Céline Gerard, Christel Péqueux, Mélanie Mestdagt, Isabelle Raymond-Letron, Claude Knauf, François Ferriere, Philippe Valet, Pierre Gourdy, Benita S Katzenellenbogen, John A Katzenellenbogen, Françoise Lenfant, Geoffrey L Greene, Jean-Michel Foidart, Jean-François Arnal

**Affiliations:** 1 INSERM U1048, Institut des Maladies Métaboliques et Cardiovasculaires, Université de Toulouse – UPSToulouse, France; 2 Institut de Recherche en Santé Environnement et Travail, IRSET, INSERM U1085, Team TREC, Biosit, Université de Rennes IRennes, France; 3 Department for Cancer Research, University of ChicagoChicago, IL, USA; 4 CNRS and Université de Toulouse, IPBSToulouse, France; 5 INSERM U1083, CNRS UMR 6214, Université d'AngersAngers, France; 6 APHP, Unité de Gynécologie Endocrinienne, Université Paris DescartesParis, France; 7 Groupe Interdisciplinaire de Génoprotéomique Appliquée (GIGA-cancer), Université de LiègeLiège, Belgique; 8 INP, ENVT, Université de ToulouseToulouse, France; 9 Departments of Molecular and Integrative Biology and Chemistry, University of Illinois at Urbana-ChampaignUrbana, IL, USA

**Keywords:** endothelium, estetrol, estrogen receptor, uterus

## Abstract

Estetrol (E_4_) is a natural estrogen with a long half-life produced only by the human fetal liver during pregnancy. The crystal structures of the estrogen receptor α (ERα) ligand-binding domain bound to 17β-estradiol (E_2_) and E_4_ are very similar, as well as their capacity to activate the two activation functions AF-1 and AF-2 and to recruit the coactivator SRC3. *In vivo* administration of high doses of E_4_ stimulated uterine gene expression, epithelial proliferation, and prevented atheroma, three recognized nuclear ERα actions. However, E_4_ failed to promote endothelial NO synthase activation and acceleration of endothelial healing, two processes clearly dependent on membrane-initiated steroid signaling (MISS). Furthermore, E_4_ antagonized E_2_ MISS-dependent effects in endothelium but also in MCF-7 breast cancer cell line. This profile of ERα activation by E_4_, uncoupling nuclear and membrane activation, characterizes E_4_ as a selective ER modulator which could have medical applications that should now be considered further.

## Introduction

Beside the well-characterized 17β-estradiol (E_2_) that is considered as the active estrogen during the estrous cycle, estriol (E_3_) and also estetrol (E_4_) are synthesized during pregnancy, but their physiological roles are essentially unknown. It is hypothesized that these two weaker estrogens could interfere with E_2_ and attenuate its actions in estrogen-sensitive tissues. Indeed, E_3_ has an affinity for estrogen receptor (ER) and a biological potency that are both tenfold lower than that of E_2_. When administered with E_2_, E_3_ can act as an antiestrogen and partially interfere with E_2_-dependent transcription (Melamed *et al*, 1997). E_4_ is viewed as a weaker estrogen, with affinity and potency 100-fold lower than those of E_2_ (Holinka & Gurpide, 1979), but its antagonistic actions are poorly defined. E_4_ shares with E_2_ and E_3_ several estrogenic activities such as uterine growth and epithelial proliferation (Holinka & Gurpide, 1979), prevention of bone demineralization (Coelingh Bennink *et al*, 2008b), inhibition of ovulation (Coelingh Bennink *et al*, 2008c), and prevention of hot flushes (Holinka *et al*, 2008).

E_4_ appears to be produced exclusively by the human fetal liver (Hagen *et al*, 1965). E_4_ also differs from E_2_ by having a long plasma half-life (about 28 h) (Visser & Coelingh Bennink, 2009), and it neither stimulates the production of nor binds to sex hormone binding globulin (SHBG) (Hammond *et al*, 2008). Because of these characteristics, E_4_ was evaluated, in combination with a progestin, as a new oral contraceptive in a phase II clinical trial (I. Duijkers I., C. Klipping C., Y. Zimmerman, L. Petit, M. Mawet, J-M. Foidart, H. Coelingh Bennink, in preparation). Very interestingly, E_4_ (up to 20 mg/day) did not elicit changes in circulating hepatic factors and thus might not increase thrombo-embolic events, which are undesirable effects of estrogen pharmaceuticals containing E_2_ or ethinyl-estradiol (EE) (C. Kluft Cornelis, Y. Zimmerman, M. Mawet Marie, C. Klipping, I. Duijkers Ingrid, L. Petit, J. Neuteboom, J-M Foidart, H. Coelingh Bennink, in preparation). Unfortunately, as previously reported (Valera *et al*, 2012), the impact of estrogen on hepatic factors is species dependent, which precludes the use of mice as an animal model to elucidate these mechanisms.

The physiological responses to estrogenic compounds are initiated by their binding to the estrogen receptors (ER), ERα and ERβ. E_4_ binds ERα with a modest preference over ERβ (Visser *et al*, 2008). ER mediates its transcriptional activity after ligand binding inducing an ordered sequence of interactions between two activation functions (AF), AF-1 and AF-2, and coactivators such as the steroid receptor coactivator (SRC) 3, a member of the p160 subfamily (McKenna & O'Malley, 2001; Metivier *et al*, 2003; Smith & O'Malley, 2004). In addition, estrogens can act through a distinctly different pathway by inducing rapid extra-nuclear activity via the activation of a pool of ERs localized at the plasma membrane, a process termed membrane-initiated steroid signaling (MISS) (Ascenzi *et al*, 2006; Wu *et al*, 2011). Although ERα MISS effects were initially also called ‘non-genomic’ effects, they can modulate ERα-dependent transcriptional activity in cultured cell models *in vitro* (La Rosa *et al*, 2012). However, thanks to a unique mouse model targeted for the ERα palmitoylation site membrane, we recently demonstrated a very contrasted involvement of MISS-mediated E_2_ action in two different tissues: the uterus in which the E_2_ response depends on ERα nuclear action and the arteries involving exclusively MISS of ERα to mediate E_2_ response (Abot *et al*, 2013; Adlanmerini *et al*, 2014).

The aim of this study was to analyze the molecular action of E_4_ using structural, *in vitro* and *in vivo* models. First, experiments were conducted to analyze the binding of E_4_ to ERα-LBD and to investigate the role of the two activation functions AF-1 and AF-2 in the transcriptional activity of E_4_ in comparison to E_2_. Second, we studied the impact of acute E_4_ treatment on gene expression and epithelial cell proliferation in uterus, which involved primarily genomic/transcriptional actions of ERα but not ERα MISS (Abot *et al*, 2013; Adlanmerini *et al*, 2014). Third, we analyzed the effect of chronic E_4_ treatment on fatty streak deposit formation at the aortic root of ovariectomized LDLr^−/−^ (Low Density Lipoprotein receptor) mice fed with an hypercholesterolemic diet. Fourth, we evaluated the effect of E_4_ on endothelial functions recognized to be dependent on MISS ERα signaling, namely acceleration of endothelial healing and activation of endothelial NO synthase (Brouchet *et al*, 2001; Toutain *et al*, 2009; Chambliss *et al*, 2010; Wu *et al*, 2011; Adlanmerini *et al*, 2014). Finally, MISS of ERα versus nuclear action after E_4_ stimulation was analyzed in the breast cancer cell line, MCF-7. The present studies reveal that high doses of E_4_ stimulated nuclear ERα actions in the uterus but E_4_ failed to promote MISS in the endothelium, and a similar profile of activation was also observed in MCF-7 cells. This profile of ERα activation indicates that E_4_ is a selective ER modulator which could have medical applications that should now be considered further, in particular in light its lesser hepatic effects in women, which could potentially reduce venous thrombo-embolic risk.

## Results

### Comparison of the ERα LBD structure, of the coactivator interaction, and of the solubility/orientation in phospholipids bilayer model membranes after E_2_ and E_4_ binding

**Figure 1:**
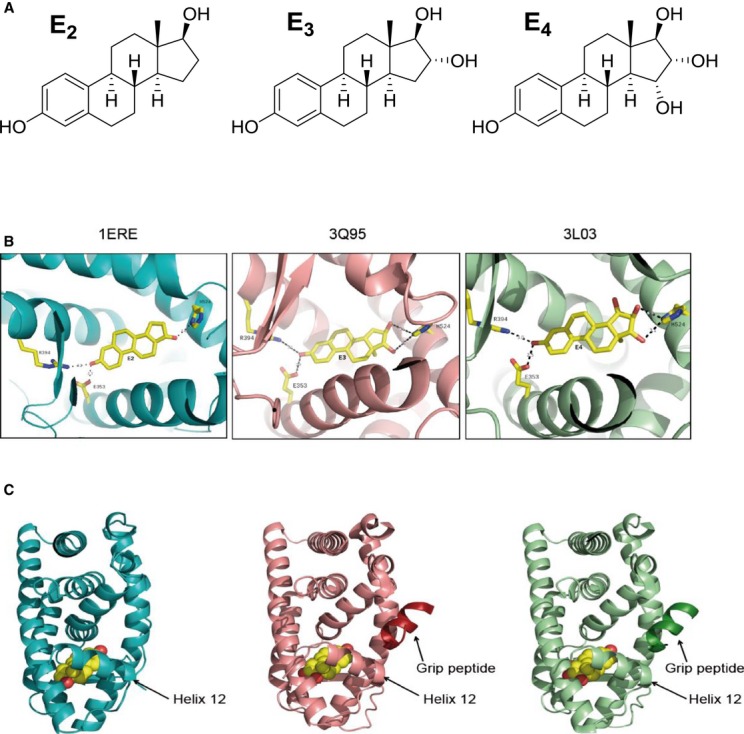
Structure of E_2_, E_3_ and E_4_ and their respective complexed structure with ERα ligand binding domain A Chemical structures of E_2_, E_3_, and E_4_. B, C Structure of ERαLBD complexed with E_2_ (blue), E_3_ (red), or E_4_ (green). Shown are ribbon diagrams of the ERαLBD monomer. Ligand-binding site (B), shown in ball-and-stick rendering of the ligands along with their interacting residues. Hydrogen bonds are shown as dotted lines. Ligand-binding domain (C) and peptide fragment of the GRIP1 coactivator protein in complex with E_3_ or E_4_ only (darker red and darker green). Ligand is represented as a space-filled model. Position of the helix 12 is indicated by an arrow.

In order to gain insight into the molecular mechanism of action of E_4,_ we first compared the crystal structures of ERα LBD complexed with E_3_ (3Q95) or E_4_ (3L03) to the published E_2_-ERα structure (1ERE) and we found all of them very similar in their overall conformation (Fig 1A and B). In addition, the two ligands are perfectly superimposable and interact equally with residues within the ligand-binding pocket (Fig 1B). The only significant difference between these structures is the altered orientation of helix 12 and the loop between helices 11 and 12 relative to that in the E_2_-ERα LBD complex (Fig 1C). However, this small difference does not prevent binding of the GRIP peptide to the E_3_- or E_4_-ERαLBD to stabilize an agonist conformation (Fig 1C). Using competitive radiometric binding assays, we found, as reported previously (Visser *et al*, 2008), that E_4_ and E_3_ bind to ERα with less affinity than E_2_ and with a small preference over ERβ (Supplementary Table S1). The binding affinity of the steroid receptor coactivator SRC3 to complexes of ligands with the ERα ligand-binding domain can be quantified by a time-resolved fluorescence resonance transfer assay (tr-FRET) (Jeyakumar *et al*, 2011). In this assay, E_3-_ERα and E_2-_ERα have essentially identical affinities for SRC3, and the affinity of E_4_-ERα, while half that of E_2_-ERα, is still in the low nanomolar range (Supplementary Fig S1 and Supplementary Table S2). Thus, as a hormonal ligand, while E_4_ has considerably lower binding affinity for ERα than E_2_, it forms a complex with this receptor that binds to a key coactivator protein, SRC3, almost as well as does the complex with E_2_.

As a consequence of its two extra hydroxyl groups, one might expect E_4_ to be less hydrophobic than E_2_ (Fig 1A); in fact, its calculated octanol–water partition coefficient (ClogP^*o/w*^) is 2.62 versus 3.78 for E_2_. Thus, we hypothesized that E_4_ would less readily partition into the plasma membrane than E_2_ (Yamamoto & Liljestrand, 2004). However, we found a similar solubility for E_2_ (˜4 mol%) and E_4_ (˜2 mol%) into palmitoyl-oleoyl-phosphatidylcholine (POPC) liposomes using nuclear magnetic resonance, indicating that their uptake is equivalent (Supplementary Fig S2A). In addition, contrary to what is described by Scheidt *et al* (2010), we found that E_2_ is in an equilibrium between two orientations in the bilayer (phenol at the lipid–water interface versus phenol within the hydrophobic core), whereas the phenol of E_4_ is oriented more predominantly toward the lipid–water interface (Supplementary Fig S2B). While unexpected, this behavior of E_4_ may be a consequence of an efficient intramolecular network of hydrogen bonds, operating among the three OH groups in the D-ring that in some way effectively suppresses their polar nature, thus allowing the D-ring to reside more comfortably in the hydrophobic core of the bilayer. In contrast, the lone 17β-OH in E_2_, which would be fully surrounded by a hydrophobic environment when in the core of the bilayer, more effectively competes with the phenolic OH for access to the aqueous interface, resulting in the two orientations of this ligand.

### Respective roles of ERα AF-1 and AF-2 in the transcription activity induced by E_4_

**Figure 2:**
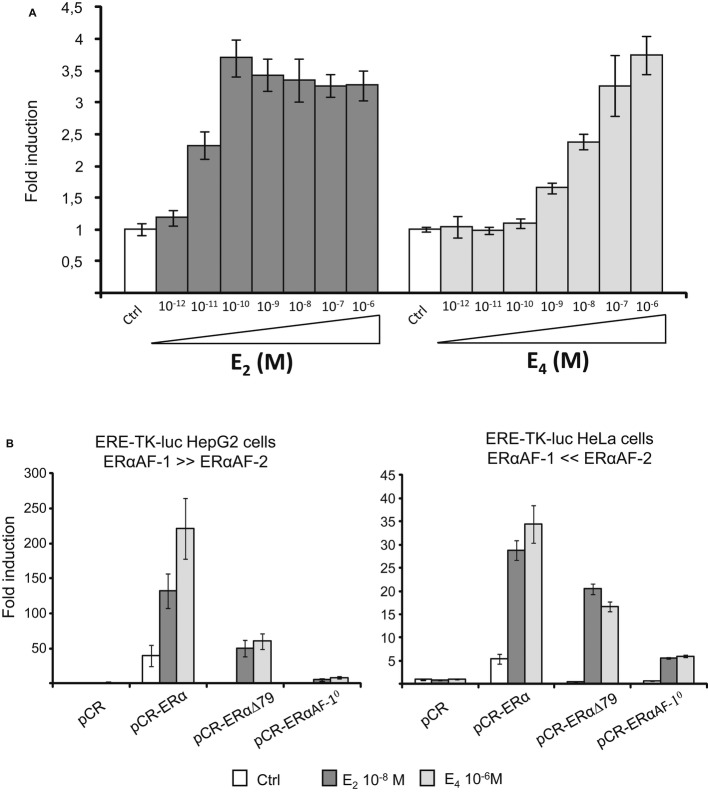
E_4_ induces ERE transcriptional activity in a cellular context-dependent manner *in vitro* in a manner similar to that of E_2_ A, B HeLa (A, B) and HepG2 (B) cells were transiently transfected with the ERE-TK-Luc reporter constructs in the presence of pCR-ERα, pCR-ERαΔ79, pCR-ERαAF-1^0^, or empty pCR vector. Cells were treated with indicated dose of E_2_ and E_4_ or vehicle (Ctrl) for 24 h. Normalized luciferase activities were expressed as fold increase above values measured with empty pCR and vehicle. Data correspond to the mean values ± SEM of at least three separate transfection experiments.

We then evaluated the ability of E_4_ to induce transcriptional activity of an estrogen-sensitive reporter gene (ERE-TK-Luc) in transient transfection assays *in vitro*. The dose–response effect of E_4_ was compared with that of E_2_ in HeLa cells transfected with an expression vector encoding the full-length ERα. E_4_ displayed a marked rightward dose–response shift compared to E_2_, requiring at least 100-fold higher hormone concentration to achieve half-maximal stimulation of the reporter gene (Fig 2A), consistent with its lower ERα binding affinity.

E_4_ modulation of activation function AF-1 and AF-2 of ERα was then evaluated in HepG2 and HeLa cell lines (Fig 2B). Whereas AF-1 is the dominant AF involved in ERα transcriptional activity in HepG2 cells, HeLa cells mediate ERα signaling mainly through AF-2 (Merot *et al*, 2004). Furthermore, cell permissiveness to either ERα AFs was determined by comparing the transcriptional activity of the full-length ERα with those of ERαΔ79 (deletion of only AF-1 box 1) and ERαAF-1^0^ (additional deletion of AF-1 box 2/3). In HepG2 cells, as is the case for E_2_, the main region involved in E_4_-induced ERα transcriptional activity is the AF-1 box 1 (ERαΔ79 versus ERα, 65% decrease of the total activity, Fig 2B), the remaining activity depending upon the AF-1 box 2/3, as expected (Huet *et al*, 2008). In contrast, the AF-1 box 1 (ERαΔ79 versus ERα) represents < 20% of the E_2_- or E_4_-induced ERα transcriptional potency in HeLa cells. These results show that a high concentration of E_4_ is able to activate gene transcription through ERα via the classical ERE mechanism. In addition, as previously described for E_2_, both AFs are involved in this action in a cell type-dependent manner.

### Impact of acute E_4_ treatment on uterine gene expression and epithelial proliferation

**Figure 3:**
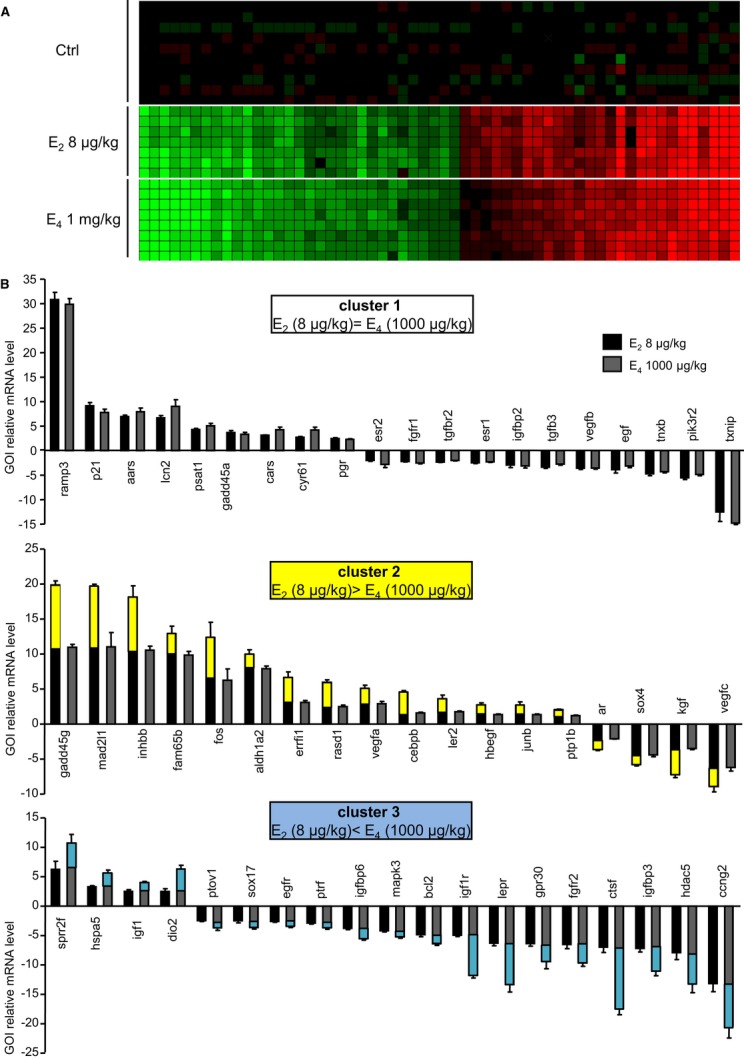
Comparison of E_2_ and E_4_ on uterine gene regulation in ovariectomized mice Seven-week-old ovariectomized C57Bl/6J mice were subcutaneously injected with vehicle (Ctrl, castor oil), E_2_ (8 μg/kg), or E_4_ (1 mg/kg) and were euthanized 6 h after treatment.
Data obtained from 96.96 Dynamic Arrays were used to generate a cluster diagram of the significant gene expression changes. Each vertical line represents a single gene. Each horizontal line represents an individual sample. Genes that were up-regulated at least twofold following E_2_ administration relative to placebo are in red, whereas down-regulated genes are in green. The color intensity indicates the degree of variation in expression.Clustering pattern of the gene whose expression is affected by E_2_ and/or E_4_.

We then assessed the transcriptional activity of E_4_ *in vivo* on the uterus in C57Bl/6J mice. We selected a set of genes known to be regulated by E_2_ in this tissue (Hewitt *et al*, 2003; Watanabe *et al*, 2003; Abot *et al*, 2013) and evaluated their expression profile in ovariectomized mice after an acute dose of each estrogen alone. Dose–response studies (E_2_: 8, 30, 80, and 200 μg/kg and E_4_: 8, 30, 80, 200, 600 μg/kg, or 1 and 10 mg/kg) indicated that most of the regulated genes reached their maximum level of induction at the lowest dose of E_2_, that is, 8 μg/kg (Table 1), and of repression, between 8 and 30 μg/kg of E_2_ (Table 2). In most cases, compared to E_2_, E_4_ required a 100-fold higher dose (i.e.*,* 1 mg/kg) to optimally activate the transcription of target genes (Table 1), although 7 of the 23 studied genes were activated at lower levels of E_4_. Concerning down-regulated genes, a dose of 80 μg/kg of E_4_ was sufficient to induce the maximal action (Table 2). Plasma analysis showed that a subcutaneous injection of 1 mg/kg E_4_ resulted in an E_4_ plasma concentration of 16,100 pg/ml after 6 h of treatment, a value close to that found for E_4_ in human fetal plasma (18,630 pg/ml). All E_2_ (8 μg/kg) target genes in the uterus were also regulated (at least twofold) by E_4_ (1 mg/kg) (Fig 3A, Tables 1 and 2) and have been distributed into three groups, according to the response to E_2_ versus E_4_ (Fig 3B). Cluster 1 represents genes similarly regulated by E_2_ at 8 μg/kg and E_4_ at 1 mg/kg doses; cluster 2 genes were found to be less regulated by E_4_ than by E_2_, and cluster 3 genes more regulated by E_4_ than by E_2_ at these doses. Yellow highlight is used to designate gene expression regulation by E_2_ that is greater than by E_4_ (Fig 3B, middle), whereas gene expression that is more regulated by the same dose of E_4_, is highlighted in blue (Fig 3B, bottom). It is noteworthy that this latter category involved mainly down-regulated genes.

**Table 1:** Seven-week-old ovariectomized C57Bl/6J mice were subcutaneously injected with vehicle (Ctrl, castor oil), 17β-estradiol (E_2_, 1, 8, 30, 80, 200 μg/kg) or estetrol (E_4_, 1, 8, 30, 80, 200 μg/kg, or 1 and 10 mg/kg) and were euthanized 6 h after treatment. mRNA levels of a set of genes from uterus that were up-regulated at least twofold by E_2_ administration relative to placebo were measured by quantitative PCR and normalized to Hprt1 expression

GOI	Dose E_2_ (µg/kg)					Dose E_4_ (µg/kg)						
	1	8	30	80	200	1	8	30	80	200	1,000	10,000
ramp3	0.81 ± 0.06	30.84 ± 1.48 *P *< 0.0001	21.71 ± 0.45 *P *<* *0.0001	22.33 ± 0.42 *P *<* *0.0001	29.04 ± 3.11 *P *<* *0.0001	0.91 ± 0.10	2.01 ± 0.36 *P *=* *0.0005	10.09 ± 0.13 *P *<* *0.0001	16.22 ± 0.21 *P *<* *0.0001	23.20 ± 0.85 *P *<* *0.0001	29.87 ± 1.17 *P *<* *0.0001	27.75 ± 2.34 *P *<* *0.0001
gadd45g	1.08 ± 0.15	19.87 ± 0.58 *P *<* *0.0001	9.05 ± 0.31 *P *<* *0.0001	6.89 ± 0.44 *P *<* *0.0001	11.96 ± 2.59 *P *<* *0.0001	0.91 ± 0.19	2.31 ± 0.49 *P *=* *0.0002	5.49 ± 0.89 *P *<* *0.0001	6.52 ± 0.89 *P *<* *0.0001	6.84 ± 0.62 *P *<* *0.0001	10.96 ± 0.42 *P *<* *0.0001	9.49 ± 0.69 *P *<* *0.0001
mad2l1	0.83 ± 0.06	19.63 ± 0.33 *P *<* *0.0001	7.69 ± 0.54 *P *<* *0.0001	5.54 ± 0.56 *P *<* *0.0001	7.59 ± 0.56 *P *<* *0.0001	0.79 ± 0.16	0.46 ± 0.17 *P *=* *0.0014	1.42 ± 0.10 *P *=* *0.0058	1.82 ± 0.23 *P *=* *0.0009	2.80 ± 0.38 *P *<* *0.0001	11.03 ± 2.03 *P *<* *0.0001	15.52 ± 0.46 *P *<* *0.0001
inhbb	0.78 ± 0.06	18.13 ± 1.62 *P *<* *0.0001	8.93 ± 0.36 *P *<* *0.0001	7.86 ± 0.41 *P *<* *0.0001	10.45 ± 1.29 *P *<* *0.0001	0.90 ± 0.03	1.44 ± 0.08 *P *=* *0.0024	5.36 ± 0.23 *P *<* *0.0001	5.76 ± 0.15 *P *<* *0.0001	6.81 ± 0.24 *P *<* *0.0001	10.55 ± 0.58 *P *<* *0.0001	11.44 ± 0.99 *P *<* *0.0001
fam65b	0.81 ± 0.04	12.92 ± 1.07 *P *<* *0.0001	10.09 ± 0.33 *P *<* *0.0001	8.03 ± 0.97 *P *<* *0.0001	9.53 ± 0.86 *P *<* *0.0001	0.97 ± 0.11	1.65 ± 0.05 *P *<* *0.0001	4.42 ± 0.58 *P *<* *0.0001	6.53 ± 0.31 *P *<* *0.0001	8.08 ± 1.01 *P *<* *0.0001	9.85 ± 0.53 *P *<* *0.0001	10.77 ± 1.31 *P *<* *0.0001
fos	0.97 ± 0.14	12.36 ± 2.19 *P *<* *0.0001	1.42 ± 0.13	1.29 ± 0.21	3.60 ± 1.39 *P *=* *0.075	0.81 ± 0.24	0.99 ± 0.22	1.64 ± 0.31	1.23 ± 0.10	3.59 ± 1.37 *P *=* *0.0182	6.26 ± 1.61 *P *=* *0.0005	2.91 ± 0.54 *P *=* *0.0001
aldh1a2	1.08 ± 0.07	9.94 ± 0.68 *P *<* *0.0001	9.29 ± 0.36 *P *<* *0.0001	9.12 ± 0.75 *P *<* *0.0001	8.61 ± 0.45 *P *<* *0.0001	1.25 ± 0.06 *P *=* *0.0241	1.07 ± 0.13	5.38 ± 0.38 *P *<* *0.0001	8.33 ± 0.36 *P *<* *0.0001	7.62 ± 0.45 *P *<* *0.0001	7.91 ± 0.39 *P *<* *0.0001	7.22 ± 0.45 *P *<* *0.0001
p21	1.01 ± 0.06	9.17 ± 0.66 *P *<* *0.0001	9.37 ± 0.34 *P *<* *0.0001	6.49 ± 0.54 *P *<* *0.0001	8.62 ± 1.04 *P *<* *0.0001	1.01 ± 0.07	1.83 ± 0.27 *P *=* *0.0002	5.57 ± 0.37 *P *<* *0.0001	6.09 ± 0.60 *P *<* *0.0001	6.89 ± 0.38 *P *<* *0.0001	7.80 ± 0.67 *P *<* *0.0001	6.33 ± 0.82 *P *<* *0.0001
aars	0.98 ± 0.05	6.95 ± 0.30 *P *<* *0.0001	10.50 ± 0.05 *P *<* *0.0001	9.00 ± 0.75 *P *<* *0.0001	9.24 ± 1.06 *P *<* *0.0001	1.04 ± 0.03	1.33 ± 0.10 *P *=* *0.0062	7.12 ± 0.04 *P *<* *0.0001	7.03 ± 0.50 *P *<* *0.0001	7.84 ± 0.73 *P *<* *0.0001	7.94 ± 0.77 *P *<* *0.0001	7.77 ± 0.62 *P *<* *0.0001
lcn2	1.04 ± 0.11	6.68 ± 0.48 *P *<* *0.0001	4.93 ± 0.51 *P *<* *0.0001	5.61 ± 0.96 *P *<* *0.0001	9.42 ± 1.18 *P *<* *0.0001	0.98 ± 0.02	0.81 ± 0.03	5.86 ± 0.56 *P *<* *0.0001	8.12 ± 0.69 *P *<* *0.0001	10.21 ± 0.90 *P *<* *0.0001	9.04 ± 1.36 *P *<* *0.0001	6.12 ± 0.42 *P *<* *0.0001
errfi1	1.06 ± 0.14	6.62 ± 0.84 *P *<* *0.0001	3.41 ± 0.18 *P *<* *0.0001	3.13 ± 0.12 *P *<* *0.0001	4.25 ± 0.96 *P *<* *0.0001	1.16 ± 0.14	1.94 ± 0.35 *P *=* *0.0002	2.86 ± 0.56 *P *<* *0.0001	2.04 ± 0.25 *P *<* *0.0001	2.45 ± 0.32 *P *<* *0.0001	3.10 ± 0.27 *P *<* *0.0001	2.30 ± 0.16 *P *<* *0.0001
sprr2f	0.89 ± 0.12	6.25 ± 1.36 *P *<* *0.0001	1.37 ± 0.09	4.48 ± 1.35 *P *=* *0.0002	4.87 ± 1.43 *P *=* *0.0007	0.98 ± 0.26	0.41 ± 0.21 *P *=* *0.0129	2.36 ± 0.55 *P *=* *0.0007	3.02 ± 0.18 *P *<* *0.0001	3.88 ± 0.80 *P *=* *0.0001	10.70 ± 1.49 *P *<* *0.0001	10.01 ± 0.75 *P *<* *0.0001
rasd1	1.13 ± 0.07	5.97 ± 0.35 *P *<* *0.0001	4.18 ± 0.19 *P *<* *0.0001	3.28 ± 0.23 *P *<* *0.0001	3.47 ± 0.19 *P *<* *0.0001	1.06 ± 0.11	1.42 ± 0.22 *P *=* *0.0134	1.61 ± 0.32 *P *=* *0.0035	1.18 ± 0.19	1.42 ± 0.37	2.49 ± 0.23 *P *<* *0.0001	3.18 ± 0.13 *P *<* *0.0001
vegfa	0.86 ± 0.10	5.04 ± 0.51 *P *<* *0.0001	4.23 ± 0.28 *P *<* *0.0001	3.06 ± 0.37 *P *<* *0.0001	3.61 ± 0.29 *P *<* *0.0001	0.97 ± 0.04	1.11 ± 0.09	2.13 ± 0.09 *P *<* *0.0001	1.43 ± 0.12 *P *=* *0.0037	1.63 ± 0.14 *P *<* *0.0001	2.91 ± 0.32 *P *<* *0.0001	3.60 ± 0.51 *P *<* *0.0001
cebpb	0.92 ± 0.03	4.54 ± 0.23 *P *<* *0.0001	2.82 ± 0.19 *P *<* *0.0001	2.37 ± 0.29 *P *<* *0.0001	2.72 ± 0.08 *P *<* *0.0001	1.21 ± 0.04	0.86 ± 0.10	1.54 ± 0.10 *P *=* *0.0011	1.08 ± 0.09	1.20 ± 0.13	1.59 ± 0.11 *P *<* *0.0001	1.87 ± 0.08 *P *<* *0.0001
psat1	1.18 ± 0.08	4.31 ± 0.23 *P *<* *0.0001	8.98 ± 0.45 *P *<* *0.0001	9.95 ± 0.70 *P *<* *0.0001	8.26 ± 1.22 *P *<* *0.0001	1.15 ± 0.09	0.81 ± 0.05	5.09 ± 0.38 *P *<* *0.0001	5.49 ± 0.45 *P *<* *0.0001	4.96 ± 0.65 *P *<* *0.0001	5.07 ± 0.51 *P *<* *0.0001	5.30 ± 0.70 *P *<* *0.0001
gadd45a	1.02 ± 0.03	3.70 ± 0.40 *P *<* *0.0001	4.88 ± 0.35 *P *<* *0.0001	4.09 ± 0.64 *P *<* *0.0001	3.77 ± 0.56 *P *<* *0.0001	0.81 ± 0.05 *P *=* *0.0436	1.21 ± 0.12	3.09 ± 0.20 *P *<* *0.0001	2.22 ± 0.24 *P *<* *0.0001	2.54 ± 0.47 *P *=* *0.0003	3.33 ± 0.40 *P *<* *0.0001	3.16 ± 0.21 *P *<* *0.0001
hspa5	1.04 ± 0.01	3.28 ± 0.19 *P *<* *0.0001	3.03 ± 0.18 *P *<* *0.0001	3.71 ± 0.31 *P *<* *0.0001	4.95 ± 0.84 *P *<* *0.0001	1.01 ± 0.01	1.18 ± 0.04	2.23 ± 0.15 *P *<* *0.0001	4.90 ± 0.32 *P *<* *0.0001	5.17 ± 0.48 *P *<* *0.0001	5.58 ± 0.54 *P *<* *0.0001	5.64 ± 0.35 *P *<* *0.0001
igf1	1.07 ± 0.03	3.27 ± 0.15 *P *<* *0.0001	2.82 ± 0.08 *P *<* *0.0001	3.52 ± 0.23 *P *<* *0.0001	3.67 ± 0.30 *P *<* *0.0001	1.11 ± 0.09	1.07 ± 0.08	3.18 ± 0.15 *P *<* *0.0001	4.19 ± 0.24 *P *<* *0.0001	3.72 ± 0.18 *P *<* *0.0001	4.01 ± 0.18 *P *<* *0.0001	5.23 ± 0.89 *P *<* *0.0001
cars	1.07 ± 0.11	3.16 ± 0.04 *P *<* *0.0001	3.55 ± 0.27 *P *<* *0.0001	3.73 ± 0.37 *P *<* *0.0001	4.26 ± 0.32 *P *<* *0.0001	0.96 ± 0.10	1.06 ± 0.08	3.19 ± 0.09 *P *<* *0.0001	3.62 ± 0.35 *P *<* *0.0001	3.47 ± 0.23 *P *<* *0.0001	4.27 ± 0.54 *P *<* *0.0001	3.96 ± 0.37 *P *<* *0.0001
cyr61	0.91 ± 0.12	2.73 ± 0.16 *P *<* *0.0001	0.96 ± 0.07	1.11 ± 0.18	2.08 ± 0.81 *P *=* *0.0560	0.73 ± 0.19	0.56 ± 0.07 *P *=* *0.0442	0.75 ± 0.12	0.94 ± 0.13	2.65 ± 0.92 *P *=* *0.0245	4.23 ± 0.57 *P *<* *0.0001	3.08 ± 0.15 *P *<* *0.0001
dio2	0.79 ± 0.06	2.49 ± 0.47 *P *=* *0.0002	2.05 ± 0.07 *P *<* *0.0001	3.67 ± 0.33 *P *<* *0.0001	4.60 ± 0.71 *P *<* *0.0001	1.03 ± 0.10	0.73 ± 0.07	1.33 ± 0.08 *P *=* *0.0036	2.68 ± 0.21 *P *<* *0.0001	4.60 ± 0.40 *P *<* *0.0001	6.30 ± 0.66 *P *<* *0.0001	5.12 ± 0.38 *P *<* *0.0001
pgr	0.87 ± 0.04	2.47 ± 0.17 *P *<* *0.0001	1.80 ± 0.03 *P *<* *0.0001	1.67 ± 0.09 *P *<* *0.0001	1.90 ± 0.07 *P *<* *0.0001	0.88 ± 0.05	1.25 ± 0.04 *P *=* *0.0163	1.58 ± 0.06 *P *<* *0.0001	1.55 ± 0.09 *P *=* *0.0002	1.74 ± 0.11 *P *<* *0.0001	2.31 ± 0.13 *P *<* *0.0001	2.20 ± 0.09 *P *<* *0.0001

Results were expressed as mean ± SEM (*n* = 4–8 mice/group). Significance of the observed effects was evaluated using Student's *t*-test. Gray highlight represents the maximum of regulation

**Table 2:** Seven-week-old ovariectomized C57Bl/6J mice were subcutaneously injected with vehicle (Ctrl, castor oil), 17β-estradiol (E_2_, 1, 8, 30, 80, 200 μg/kg) or estetrol (E_4_, 1, 8, 30, 80, 200 μg/kg, or 1 and 10 mg/kg) and were euthanized 6 h after treatment. mRNA levels of a set of genes from uterus that were down-regulated at least twofold by E_2_ administration relative to placebo were measured by quantitative PCR and normalized to Hprt1 expression

GOI	Dose E_2_ (µg/kg)					Dose E_4_ (µg/kg)						
	1	8	30	80	200	1	8	30	80	200	1,000	10,000
esr2	0.92 ± 0.09	0.50 ± 0.04 *P *=* *0.0033	0.32 ± 0.05 *P *=* *0.0018	0.34 ± 0.04 *P *=* *0.0006	0.56 ± 0.08 *P *=* *0.0077	0.90 ± 0.22	0.41 ± 0.20 *P *=* *0.0056	0.32 ± 0.05 *P *=* *0.0019	0.13 ± 0.04 *P *<* *0.0001	0.26 ± 0.08 *P *<* *0.0001	0.36 ± 0.08 *P *<* *0.0001	0.34 ± 0.06 *P *=* *0.0006
fgfr1	0.98 ± 0.01	0.45 ± 0.02 *P *<* *0.0001	0.40 ± 0.03 *P *<* *0.0001	0.33 ± 0.04 *P *<* *0.0001	0.39 ± 0.03 *P *<* *0.0001	1.10 ± 0.04	0.95 ± 0.08	0.68 ± 0.05 *P *=* *0.0020	0.44 ± 0.02 *P *<* *0.0001	0.42 ± 0.03 *P *<* *0.0001	0.40 ± 0.03 *P *<* *0.0001	0.44 ± 0.05 *P *<* *0.0001
tgfbr2	0.90 ± 0.05	0.43 ± 0.01 *P *<* *0.0001	0.33 ± 0.02 *P *<* *0.0001	0.32 ± 0.02 *P *<* *0.0001	0.39 ± 0.03 *P *<* *0.0001	0.85 ± 0.04	0.87 ± 0.02	0.54 ± 0.02 *P *=* *0.0007	0.47 ± 0.02 *P *<* *0.0001	0.51 ± 0.03 *P *<* *0.0001	0.50 ± 0.02 *P *<* *0.0001	0.57 ± 0.07 *P *=* *0.0005
esr1	0.98 ± 0.03	0.41 ± 0.03 *P *<* *0.0001	0.27 ± 0.01 *P *<* *0.0001	0.29 ± 0.02 *P *<* *0.0001	0.38 ± 0.02 *P *<* *0.0001	0.90 ± 0.03	1.04 ± 0.04	0.67 ± 0.02 *P *=* *0.0101	0.44 ± 0.02 *P *<* *0.0001	0.44 ± 0.02 *P *<* *0.0001	0.44 ± 0.02 *P *<* *0.0001	0.41 ± 0.02 *P *<* *0.0001
ptov1	0.56 ± 0.08 *P *=* *0.0028	0.40 ± 0.03 *P *<* *0.0001	0.20 ± 0.02 *P *<* *0.0001	0.21 ± 0.02 *P *<* *0.0001	0.29 ± 0.05 *P *<* *0.0001	0.65 ± 0.05 *P *=* *0.0110	0.56 ± 0.03 *P *=* *0.0019	0.25 ± 0.03 *P *<* *0.0001	0.14 ± 0.02 *P *<* *0.0001	0.24 ± 0.02 *P *<* *0.0001	0.27 ± 0.03 *P *<* *0.0001	0.28 ± 0.03 *P *<* *0.0001
sox17	1.01 ± 0.05	0.40 ± 0.06 *P *<* *0.0001	0.43 ± 0.02 *P *=* *0.0006	0.35 ± 0.04 *P *<* *0.0001	0.40 ± 0.02 *P *<* *0.0001	0.90 ± 0.08	1.02 ± 0.04	0.56 ± 0.04 *P *=* *0.0044	0.34 ± 0.01 *P *<* *0.0001	0.37 ± 0.01 *P *<* *0.0001	0.27 ± 0.02 *P *<* *0.0001	0.28 ± 0.03 *P *<* *0.0001
egfr	1.19 ± 0.04	0.38 ± 0.02 *P *<* *0.0001	0.53 ± 0.02 *P *=* *0.0062	0.52 ± 0.06 *P *=* *0.0022	0.63 ± 0.05 *P *=* *0.0064	1.53 ± 0.08 *P *=* *0.0011	0.77 ± 0.02	0.41 ± 0.03 *P *=* *0.0013	0.29 ± 0.02 *P *<* *0.0001	0.29 ± 0.03 *P *<* *0.0001	0.30 ± 0.03 *P *<* *0.0001	0.43 ± 0.04 *P *=* *0.0004
ptrf	0.90 ± 0.03	0.34 ± 0.02 *P *<* *0.0001	0.31 ± 0.02 *P *<* *0.0001	0.29 ± 0.02 *P *<* *0.0001	0.35 ± 0.02 *P *<* *0.0001	1.13 ± 0.04	0.79 ± 0.01 *P *=* *0.0258	0.39 ± 0.03 *P *<* *0.0001	0.25 ± 0.01 *P *<* *0.0001	0.25 ± 0.01 *P *<* *0.0001	0.28 ± 0.02 *P *<* *0.0001	0.29 ± 0.03 *P *<* *0.0001
igfbp2	1.07 ± 0.16	0.34 ± 0.06 *P *<* *0.0001	0.64 ± 0.13 *P *=* *0.0122	0.63 ± 0.11 *P *=* *0.0056	0.58 ± 0.06 *P *=* *0.0005	2.15 ± 0.42 *P *=* *0.0002	2.70 ± 1.84	1.15 ± 0.62	0.38 ± 0.03 *P *<* *0.0001	0.43 ± 0.11 *P *<* *0.0001	0.32 ± 0.04 *P *<* *0.0001	0.36 ± 0.10 *P *<* *0.0001
tgfb3	1.14 ± 0.07	0.30 ± 0.02 *P *<* *0.0001	0.47 ± 0.03 *P *=* *0.0002	0.41 ± 0.03 *P *<* *0.0001	0.41 ± 0.04 *P *<* *0.0001	1.06 ± 0.05	0.86 ± 0.06	0.31 ± 0.01 *P *<* *0.0001	0.26 ± 0.01 *P *<* *0.0001	0.25 ± 0.01 *P *<* *0.0001	0.37 ± 0.03 *P *<* *0.0001	0.53 ± 0.04 *P *=* *0.0002
vegfb	0.93 ± 0.02	0.28 ± 0.02 *P *<* *0.0001	0.22 ± 0.03 *P *<* *0.0001	0.20 ± 0.03 *P *<* *0.0001	0.27 ± 0.01 *P *<* *0.0001	1.06 ± 0.06	0.81 ± 0.03	0.40 ± 0.04 *P *=* *0.0003	0.23 ± 0.01 *P *<* *0.0001	0.26 ± 0.01 *P *<* *0.0001	0.28 ± 0.02 *P *<* *0.0001	0.32 ± 0.02 *P *<* *0.0001
ar	1.07 ± 0.04	0.28 ± 0.01 *P *<* *0.0001	0.26 ± 0.01 *P *<* *0.0001	0.31 ± 0.02 *P *<* *0.0001	0.37 ± 0.02 *P *<* *0.0001	0.99 ± 0.03	1.01 ± 0.04	0.51 ± 0.02 *P *=* *0.0003	0.41 ± 0.01 *P *<* *0.0001	0.45 ± 0.01 *P *<* *0.0001	0.48 ± 0.01 *P *<* *0.0001	0.51 ± 0.02 *P *<* *0.0001
igfbp6	0.80 ± 0.03	0.26 ± 0.02 *P *<* *0.0001	0.19 ± 0.01 *P *<* *0.0001	0.19 ± 0.02 *P *<* *0.0001	0.19 ± 0.01 *P *<* *0.0001	0.81 ± 0.03	0.75 ± 0.04	0.34 ± 0.01 *P *=* *0.0003	0.21 ± 0.01 *P *<* *0.0001	0.21 ± 0.01 *P *<* *0.0001	0.18 ± 0.01 *P *<* *0.0001	0.19 ± 0.02 *P *<* *0.0001
egf	0.93 ± 0.06	0.26 ± 0.05 *P *<* *0.0001	0.38 ± 0.02 *P *=* *0.0003	0.34 ± 0.02 *P *<* *0.0001	0.40 ± 0.06 *P *<* *0.0001	1.20 ± 0.10	0.61 ± 0.04 *P *=* *0.0036	0.29 ± 0.02 *P *<* *0.0001	0.22 ± 0.02 *P *<* *0.0001	0.33 ± 0.01 *P *<* *0.0001	0.32 ± 0.03 *P *<* *0.0001	0.35 ± 0.02 *P *<* *0.0001
mapk3	1.11 ± 0.03	0.24 ± 0.01 *P *<* *0.0001	0.21 ± 0.02 *P *<* *0.0001	0.21 ± 0.01 *P *<* *0.0001	0.22 ± 0.01 *P *<* *0.0001	1.13 ± 0.04	0.78 ± 0.02 *P *=* *0.0497	0.34 ± 0.02 *P *<* *0.0001	0.19 ± 0.01 *P *<* *0.0001	0.19 ± 0.01 *P *<* *0.0001	0.19 ± 0.01 *P *<* *0.0001	0.19 ± 0.01 *P *<* *0.0001
tnxb	0.98 ± 0.02	0.21 ± 0.02 *P *<* *0.0001	0.30 ± 0.01 *P *<* *0.0001	0.27 ± 0.03 *P *<* *0.0001	0.30 ± 0.02 *P *<* *0.0001	1.04 ± 0.02	0.89 ± 0.03	0.31 ± 0.01 *P *<* *0.0001	0.17 ± 0.01 *P *<* *0.0001	0.19 ± 0.01 *P *<* *0.0001	0.23 ± 0.01 *P *<* *0.0001	0.24 ± 0.03 *P *<* *0.0001
bcl2	1.10 ± 0.01	0.21 ± 0.02 *P *<* *0.0001	0.24 ± 0.02 *P *<* *0.0001	0.23 ± 0.01 *P *<* *0.0001	0.23 ± 0.01 *P *<* *0.0001	1.12 ± 0.08	0.59 ± 0.03 *P *=* *0.0002	0.17 ± 0.02 *P *<* *0.0001	0.10 ± 0.01 *P *<* *0.0001	0.12 ± 0.01 *P *<* *0.0001	0.16 ± 0.01 *P *<* *0.0001	0.18 ± 0.01 *P *<* *0.0001
igf1r	1.00 ± 0.04	0.20 ± 0.01 *P *<* *0.0001	0.15 ± 0.01 *P *<* *0.0001	0.11 ± 0.01 *P *<* *0.0001	0.12 ± 0.01 *P *<* *0.0001	1.04 ± 0.02	0.59 ± 0.01 *P *=* *0.0005	0.21 ± 0.02 *P *<* *0.0001	0.09 ± 0.01 *P *<* *0.0001	0.08 ± 0.01 *P *<* *0.0001	0.09 ± 0.01 *P *<* *0.0001	0.09 ± 0.01 *P *<* *0.0001
pik3r2	0.83 ± 0.02	0.18 ± 0.01 *P *<* *0.0001	0.14 ± 0.01 *P *<* *0.0001	0.15 ± 0.02 *P *<* *0.0001	0.20 ± 0.02 *P *<* *0.0001	0.84 ± 0.05	0.82 ± 0.03	0.31 ± 0.02 *P *<* *0.0001	0.16 ± 0.01 *P *<* *0.0001	0.20 ± 0.02 *P *<* *0.0001	0.21 ± 0.01 *P *<* *0.0001	0.18 ± 0.02 *P *<* *0.0001
sox4	0.83 ± 0.03	0.17 ± 0.01 *P *<* *0.0001	0.16 ± 0.01 *P *<* *0.0001	0.16 ± 0.02 *P *<* *0.0001	0.20 ± 0.01 *P *<* *0.0001	0.73 ± 0.04	0.61 ± 0.11 *P *=* *0.0139	0.19 ± 0.02 *P *<* *0.0001	0.13 ± 0.01 *P *<* *0.0001	0.18 ± 0.01 *P *<* *0.0001	0.23 ± 0.01 *P *<* *0.0001	0.22 ± 0.02 *P *<* *0.0001
lepr	1.13 ± 0.03	0.16 ± 0.01 *P *<* *0.0001	0.17 ± 0.01 *P *<* *0.0001	0.12 ± 0.02 *P *<* *0.0001	0.13 ± 0.01 *P *<* *0.0001	1.26 ± 0.04 *P *=* *0.462	0.79 ± 0.06	0.28 ± 0.03 *P *=* *0.0001	0.12 ± 0.01 *P *<* *0.0001	0.10 ± 0.01 *P *<* *0.0001	0.07 ± 0.01 *P *<* *0.0001	0.07 ± 0.01 *P *<* *0.0001
gpr30	1.04 ± 0.04	0.16 ± 0.01 *P *<* *0.0001	0.20 ± 0.04 *P *<* *0.0001	0.16 ± 0.01 *P *<* *0.0001	0.17 ± 0.01 *P *<* *0.0001	1.10 ± 0.05	0.74 ± 0.08	0.23 ± 0.03 *P *<* *0.0001	0.11 ± 0.01 *P *<* *0.0001	0.11 ± 0.01 *P *<* *0.0001	0.11 ± 0.01 *P *<* *0.0001	0.12 ± 0.02 *P *<* *0.0001
fgfr2	1.25 ± 0.07	0.15 ± 0.02 *P *<* *0.0001	0.17 ± 0.01 *P *=* *0.0001	0.17 ± 0.04 *P *<* *0.0001	0.13 ± 0.01 *P *<* *0.0001	1.03 ± 0.05	0.95 ± 0.02	0.25 ± 0.03 *P *=* *0.0003	0.13 ± 0.01 *P *<* *0.0001	0.12 ± 0.01 *P *<* *0.0001	0.10 ± 0.01 *P *<* *0.0001	0.12 ± 0.02 *P *<* *0.0001
ctsf	1.02 ± 0.02	0.14 ± 0.02 *P *<* *0.0001	0.11 ± 0.01 *P *<* *0.0001	0.08 ± 0.01 *P *<* *0.0001	0.10 ± 0.01 *P *<* *0.0001	1.03 ± 0.04	0.71 ± 0.04 *P *=* *0.0068	0.19 ± 0.02 *P *<* *0.0001	0.07 ± 0.01 *P *<* *0.0001	0.07 ± 0.01 *P *<* *0.0001	0.06 ± 0.01 *P *<* *0.0001	0.06 ± 0.01 *P *<* *0.0001
kgf	1.28 ± 0.15	0.14 ± 0.01 *P *<* *0.0001	0.16 ± 0.01 *P *<* *0.0001	0.18 ± 0.02 *P *<* *0.0001	0.24 ± 0.02 *P *<* *0.0001	1.12 ± 0.07	1.02 ± 0.04	0.41 ± 0.02 *P *=* *0.0007	0.31 ± 0.01 *P *<* *0.0001	0.36 ± 0.03 *P *<* *0.0001	0.29 ± 0.01 *P *<* *0.0001	0.28 ± 0.020 *P *<* *0.0001
igfbp3	1.10 ± 0.06	0.14 ± 0.01 *P *<* *0.0001	0.15 ± 0.01 *P *=* *0.0003	0.13 ± 0.02 *P *<* *0.0001	0.13 ± 0.02 *P *<* *0.0001	1.18 ± 0.11	0.65 ± 0.05 *P *=* *0.0379	0.21 ± 0.01 *P *=* *0.0005	0.12 ± 0.01 *P *<* *0.0001	0.10 ± 0.01 *P *<* *0.0001	0.09 ± 0.01 *P *<* *0.0001	0.11 ± 0.01 *P *<* *0.0001
hdac5	0.74 ± 0.06 *P *=* *0.0438	0.13 ± 0.02 *P *<* *0.0001	0.09 ± 0.01 *P *<* *0.0001	0.08 ± 0.01 *P *<* *0.0001	0.09 ± 0.01 *P *<* *0.0001	0.88 ± 0.07	0.58 ± 0.05 *P *=* *0.0023	0.19 ± 0.03 *P *<* *0.0001	0.06 ± 0.01 *P *<* *0.0001	0.07 ± 0.01 *P *<* *0.0001	0.08 ± 0.01 *P *<* *0.0001	0.06 ± 0.01 *P *<* *0.0001
vegfc	1.06 ± 0.08	0.11 ± 0.01 *P *<* *0.0001	0.11 ± 0.01 *P *<* *0.0001	0.11 ± 0.01 *P *<* *0.0001	0.13 ± 0.01 *P *<* *0.0001	1.02 ± 0.05	0.78 ± 0.06	0.30 ± 0.01 *P *<* *0.0001	0.18 ± 0.01 *P *<* *0.0001	0.15 ± 0.01 *P *<* *0.0001	0.16 ± 0.01 *P *<* *0.0001	0.16 ± 0.01 *P *<* *0.0001
txnip	1.29 ± 0.05 *P *=* *0.0246	0.08 ± 0.01 *P *<* *0.0001	0.09 ± 0.01 *P *<* *0.0001	0.08 ± 0.01 *P *<* *0.0001	0.10 ± 0.01 *P *<* *0.0001	1.32 ± 0.06 *P *=* *0.0160	0.68 ± 0.03 *P *=* *0.0144	0.15 ± 0.01 *P *<* *0.0001	0.09 ± 0.01 *P *<* *0.0001	0.09 ± 0.01 *P *<* *0.0001	0.07 ± 0.01 *P *<* *0.0001	0.07 ± 0.01 *P *<* *0.0001
ccng2	0.99 ± 0.03	0.08 ± 0.01 *P *<* *0.0001	0.08 ± 0.01 *P *<* *0.0001	0.06 ± 0.01 *P *<* *0.0001	0.06 ± 0.01 *P *<* *0.0001	1.11 ± 0.02	0.58 ± 0.03 *P *=* *0.0003	0.10 ± 0.02 *P *<* *0.0001	0.04 ± 0.01 *P *<* *0.0001	0.04 ± 0.01 *P *<* *0.0001	0.05 ± 0.01 *P *<* *0.0001	0.06 ± 0.01 *P *<* *0.0001

Results were expressed as mean ± SEM (*n* = 4–8 mice/group). Significance of the observed effects was evaluated using Student's *t*-test. Gray highlight represents the maximum of regulation

**Figure 4:**
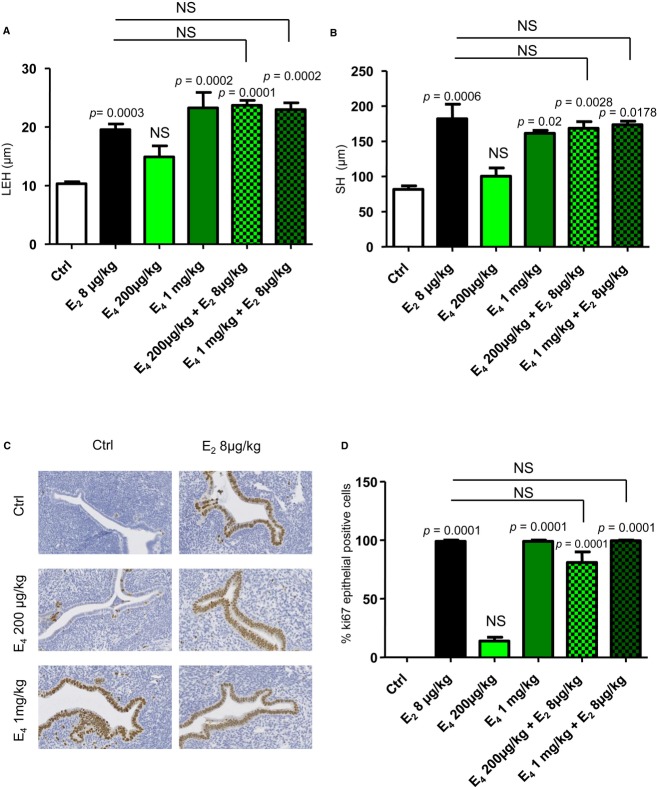
Comparison of E_2_ and E_4_ on uterine histological parameters and epithelial proliferation Seven-week-old ovariectomized C57Bl/6J mice were injected subcutaneously with vehicle (Ctrl, castor oil), E_2_ (8 μg/kg), and/or E_4_ (200 μg/kg or 1 mg/kg) and were euthanized 24 h after treatment. A, B Luminal epithelial height (LEH) (A) and stromal height (SH) (B) were measured. C, D Representative (C) and quantification (D) of Ki-67 detection in transverse uterus sections (scale bar = 50 μm). Data information: Results are expressed as mean ± SEM. To test the respective roles of each treatment, a one-way ANOVA was performed and a Bonferroni's multiple comparison test (*n* = 4–6 mice/group).

We next examined the relationship between gene regulation patterns and uterotrophic effects of E_2_ versus E_4_, noting histological changes and uterine epithelial cell proliferation. Luminal epithelial height (LEH) and stromal height (SH) were significantly and similarly increased with E_2_ (8 μg/kg) and E_4_ (1 mg/kg) 24 h after subcutaneous administration (Fig 4), without significant effects for doses of E_4_ < 1 mg/kg (Fig 4A and B, and Supplementary Fig S3A and B). Accordingly, a maximal induction of epithelial proliferation, detected by Ki-67 nuclear staining (Fig 4C and D), was observed in mice treated with either E_2_ 8 μg/kg or E_4_ 1 mg/kg alone. Lower doses of E_4_ elicited moderate to minor epithelial proliferation (Supplementary Fig S3C and D). To further analyze the interactions between E_4_ and E_2_ on ERα transcriptional activity, we then studied the effect of their combined impact on uterus. E_2_ (8 μg/kg) and E_4_ (given at either 200 μg/kg or 1 mg/kg) were co-administrated, and gene expression in the uterus was analyzed 6 h later. As shown in the Supplementary Fig S4, the gene expression profile of the E_2_–E_4_ combination was similar to that elicited by E_2_ alone for most of the genes (cluster 1). In some cases an intermediate response was observed using co-administration of E_2_–E_4_ compared to E_2_ alone (cluster 2), probably due to the lower potency of E_4_ (1 mg/kg) than those of E_2_ to induce maximal gene regulation for these genes (Fig 3, middle panel). Importantly, the histological changes and uterine epithelial cell proliferation induced by E_2_ (8 μg/kg) and E_4_ (200 μg/kg or 1 mg/kg) co-treatment did not differ from those elicited by E_2_ (8 μg/kg) alone (Fig 4). Taken together, these results demonstrate that E_4_ acts as a less potent estrogen on both gene expression and epithelial proliferation in the uterus, close to results obtained previously in rat uterus (Holinka & Gurpide, 1979).

### E_4_ induces an atheroprotective effect in an ERα-dependent manner

**Figure 5:**
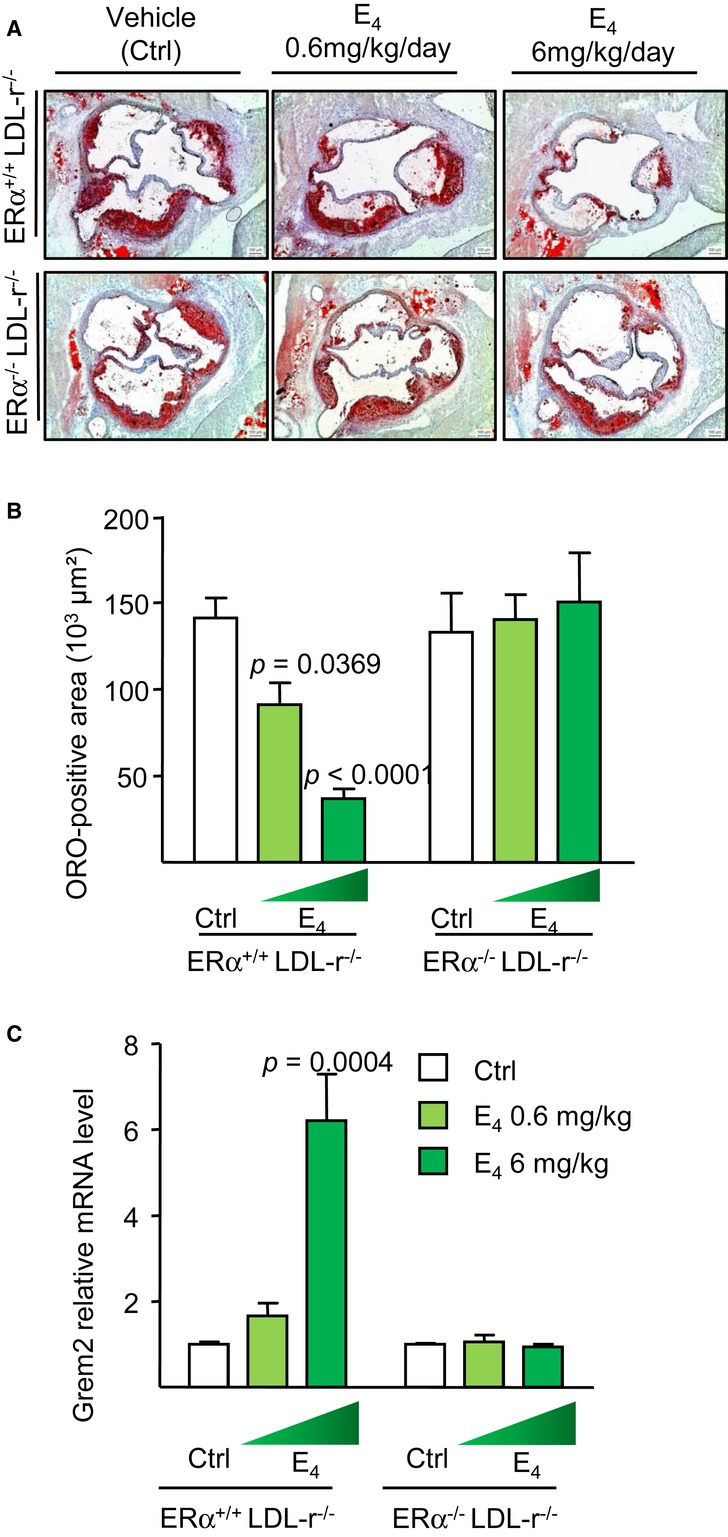
E_4_ prevents aortic sinus lipid deposition in hypercholesterolemic mice Four-week-old ovariectomized ERα^+/+^LDL-r^−/−^ or ERα^−/−^LDL-r^−/−^ mice were switched to atherogenic diet from the age of 6–18 weeks added with placebo (Ctrl) or E_4_ (0.6 or 6 mg/kg/day). A, B Representative micrographs of Oil red-O (ORO) lipid-stained cryosections of the aortic sinus (A) and quantification of lipid deposition (B) are represented. C Gremlin 2 (*Grem2*) mRNA level from aorta of these mice was quantified by qPCR and normalized to *Tpt1* mRNA levels. Result was expressed according to the level in aorta from placebo set as 1. Data information: Results are expressed as mean ± SEM. Significance of the observed effects was evaluated using one-way or two-way ANOVA followed by Bonferroni's *post hoc* test (*n* = 4–8 mice/group).

Since estrogens exert many beneficial effects on the arteries (Arnal *et al*, 2012), we assessed the impact of E_4_ on the prevention of atheroma. For this aim, we examined lipid deposition at the aortic sinus from ERα^+/+^LDLr^−/−^ or ERα^−/−^LDLr^−/−^ (Low Density Lipoprotein receptor) mice fed a high-cholesterol diet supplemented or not with E_4_ (0.6 and 6 mg/kg/day), a well-recognized model to study atheroprotective effects of estrogens (Mallat & Tedgui, 2007; Weber *et al*, 2008). E_4_ dose-dependently prevented lipid deposition in ovariectomized ERα^+/+^LDLr^−/−^ mice (Fig 5A and B), decreasing the atheroma deposit by up to 80%, a level of protection similar to that obtained using a high dose of E_2_ (80 μg/kg/jour) (Billon-Gales *et al*, 2009). As previously observed with E_2_, this effect was completely abolished in ERα^−/−^LDLr^−/−^ mice, indicating that ERα is necessary to mediate the atheroprotective effect of E_4_ (Fig 5A and B). Interestingly, expression of the most strongly induced gene by E_2_ in the aorta, Gremlin 2 (Grem2) (Schnoes *et al*, 2008) was found to be regulated by the highest dose of E_4_ in ERα^+/+^LDLr^−/−^, but not in ERα^−/−^LDLr^−/−^ mice (Fig 5C), emphasizing another aspect of the ERα-dependent nuclear regulation by E_4_.

As previously observed with E_2_ (Billon-Gales *et al*, 2009), E_4_ (6 mg/kg/day) decreased total plasma cholesterol in ERα^+/+^LDLr^−/−^ but not in ERα^−/−^LDLr^−/−^ mice. However, in contrast to the action of E_2_, no change of HDL cholesterol level was observed in E_4_ treated mice (Table 3). As expected from the acute dose experiments, a dose-dependent uterine hypertrophy was observed in mice receiving E_4_ chronically, and this effect was totally abolished in ERα^−/−^LDLr^−/−^ mice, further demonstrating the crucial role of ERα in E_4_ uterotrophic activity (Table 3).

**Table 3:** Effect of E_4_ (0.6 or 6 mg/kg/day) treatment on body weight, uterine weight, plasma lipid concentrations, and Oil-red O (ORO) positive area at the aortic sinus in 18-week-old ERα^+/+^LDLr^−/−^ or ERα^−/−^LDLr^−/−^ mice

	ERα^+/+^LDLr^−/−^			ERα^−/−^ LDLr^−/−^			*P*, two-factor ANOVA		
	Ctrl (*n* = 10)	E_4_ 0.6 mg/kg/day (*n* = 9)	E_4_ 6 mg/kg/day (*n* = 7)	Ctrl (*n* = 7)	E_4_ 0.6 mg/kg/day (*n* = 8)	E_4_ 6 mg/kg/day (*n* = 4)	Genotype	E_4_	Interaction
Body weight (g)	21.5 ± 0.9	18.7 ± 0.6 *P *=* *0.0187	16.2 ± 0.3 *P *<* *0.0001	20.9 ± 0.8	23.2 ± 0.5	22.0 ± 1.1	–	–	*P *=* *0.0004
Uterine weight (mg)	6 ± 1	31 ± 3 *P *<* *0.0001	71 ± 7 *P *<* *0.0001	3 ± 1	4 ± 1	6 ± 1	–	–	*P *=* *0.0001
Total Chol. (mg/dl)	1152.8 ± 142.2	868.4 ± 154.6	552.6 ± 44.0 *P *=* *0.0065	1102.2 ± 205.3	1356.5 ± 124.5	1633.3 ± 276.3	–	–	*P *=* *0.0052
HDL Chol. (mg/dl)	62.3 ± 9.8	77.2 ± 15.1	63.7 ± 4.9	56.9 ± 14.9	61.9 ± 6.2	82.6 ± 24.1	NS	NS	NS
ORO area (×10^3^ µm^2^)	141 ± 11	91 ± 13 *P *=* *0.0369	37 ± 5 *P *<* *0.0001	133 ± 23	140 ± 14	151 ± 28	–	^-^	*P *=* *0.0028

Results were expressed as mean ± SEM. Significance of the observed effects was evaluated using two-way ANOVA. When an interaction was observed between the 2 factors, effect of E_4_ treatment was studied in each genotype using a Bonferroni's *post hoc* test (*n* = 4–8 mice/group)

### E_4_ fails to increase endothelial NO production and to accelerate endothelial healing

**Figure 6:**
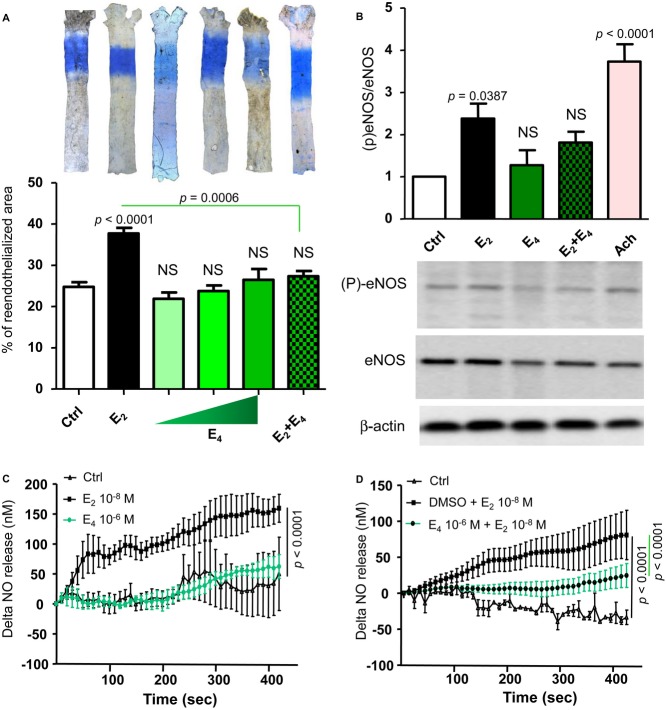
E_4_ fails to accelerate reendothelialization and to increase NO production Seven-week-old ovariectomized C57Bl/6J mice were given placebo (Ctrl), E_2_ (80 μg/kg/day) or E_4_ (0.3–6 mg/kg/day), or E_2_ (80 μg/kg/day) + E_4_ (6 mg/kg/day) for 2 weeks.
Electric injury was applied to the distal part (3 mm precisely) of the common carotid artery, and the endothelial regeneration process was evaluated 3 days postinjury. Quantification of the reendothelialized area evaluated by Evans blue staining, and results were expressed as mean ± SEM (*n* = 7–23 mice per group). Significance of the observed effects was evaluated using one-way ANOVA followed by Bonferroni's *post hoc* test.Quantification expressed as mean ± SEM (*n* = 7 mice per group, upper panel) and representative Western blot (lower panel) of phospho-eNOS/eNOS abundance in isolated aortae treated by E_2_ (10^−8^ M), E_4_ (10^−6^ M), combination of both E_2_ and E_4_ or acetylcholine (Ach) used as a positive control during 30 min. Significance of the observed effects was evaluated using one-way ANOVA followed by Bonferroni's *post hoc* test (*n* = 8 mice/group).Representative trace of *ex vivo* amperometric measurements of NO release of aortae from 10- to 12-week-old C57Bl/6J mice exposed to E_2_ (10^−8^ M) or E_4_ (10^−6^ M) during 5 min.For cotreatment experiment, E_4_ (10^−6^ M) or vehicle (DMSO) was pre-incubated during 10 min prior to E_2_ (10^−8^ M) treatment. To test the respective roles of each treatment, a one-way ANOVA was performed followed by a Bonferroni's *post hoc* test. Source data are available online for this figure.

We then tested the effect of E_4_ on two other important vasculoprotective actions of estrogens, namely the acceleration of reendothelialization (Brouchet *et al*, 2001; Chambliss *et al*, 2010) and activation of eNOS (Wu *et al*, 2011), both of which are known to involve ERα MISS in the endothelium (Adlanmerini *et al*, 2014). First, although E_2_ promoted endothelial healing in the model of carotid artery electric injury, no effect was observed with E_4_, regardless of the dose employed (0.3, 1 or 6 mg/kg/day) (Fig 6A). Second, we tested the effect of E_4_ on eNOS activation in aortae by measuring eNOS phosphorylation (Fig 6B) and NO production using a NO-specific amperometric probe. Whereas E_2_ (10^−8^ M) rapidly and nicely induced eNOS phosphorylation (Fig 6B) and NO production (Fig 6C) in aortae, E_4_ (10^−6^ M) failed to produce these effects (Fig 6B and C). Together, these results suggest that E_4_ is not able to elicit two major endothelial actions known to be MISS ERα dependent, namely acceleration of reendothelialization and activation of eNOS.

The fact that E_4_ failed to elicit responses that are mediated via membrane ERα raises the question of whether this is due to the failure of E_4_ to bind to membrane ERα or the failure of membrane ERα to become activated by E_4_ binding, in which case E_4_ would be expected to have antagonist activity on this signaling pathway. To address this question, we first co-administrated E_4_ (6 mg/kg/day) and E_2_ (80 μg/kg/day), and found that this combination failed to accelerate endothelial healing (Fig 6A). Then, we tested the effect of E_2_ (10^−8 ^M) on NO production by aortae *ex vivo* exposed to E_4_ (10^−6 ^M) 10 min before, and we found that E_4_ inhibited the stimulatory action of E_2_ (Fig 6D). Accordingly, the combination of E_2_ (10^−8 ^M) and E_4_ (10^−6 ^M) did not stimulate eNOS phosphorylation in aortae (Fig 6B). Altogether, E_4_ is not only devoid of ERα MISS in the endothelium, but E_4_ is also able to partially antagonize these E_2_ MISS effects.

### E_4_ promotes ERα-*src* interaction less efficiently than does E_2_ but induces similar ERE-dependent transcriptional activity in MCF-7

**Figure 7:**
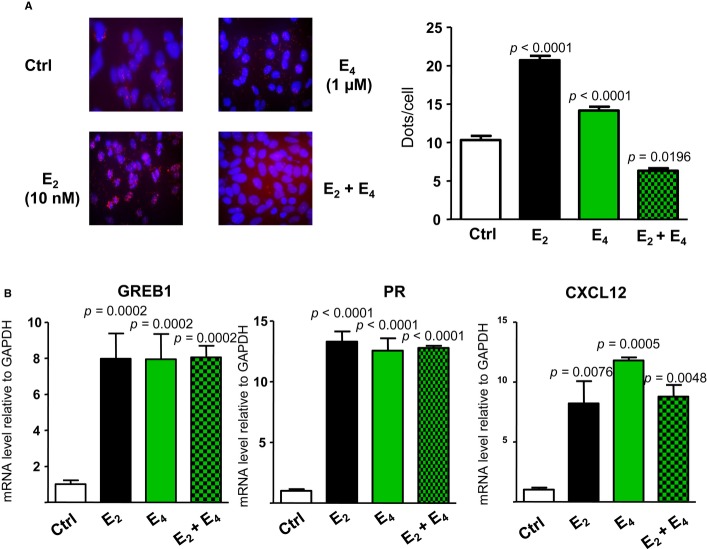
E_4_ promotes ERα-*src* interaction less efficiently than does E_2_ but induces similar ERE-dependent transcriptional activity in MCF-7 MCF-7 cells were grown in medium containing 2.5% charcoal-stripped serum with vehicle or with E_2_ (10^−8^ M), E_4_ (10^−6^ M) or in combination for 5 min. After fixation, *in situ* PLA for ERα-Src dimers was performed with ERα- and Src-specific antibodies. The detected dimers are represented by red dots, and the nuclei were counterstained with DAPI (blue). Quantification of the number of signals per cell was performed by computer-assisted analysis as reported in the Materials and Methods section. Values correspond to the mean ± SEM of at least three separate experiments, and columns with different superscripts differ significantly using Student's *t*-test.mRNA level of the indicated gene from MCF-7 cells treated with vehicle, E_2_ (10^−8^ M), E_4_ (10^−6^ M) or combined treatment and analyzed after 24 h by qPCR. Values correspond to the mean ± SD of at least three separate experiments. To test the respective roles of each treatment, a one-way ANOVA was performed and a Bonferroni's multiple comparison test.

Finally, we approached the impact of E_4_ on ERα MISS in the breast cancer cell line, MCF-7. We failed to detect reliably the activation of MAPK by E_2_, in agreement with some authors (Gaben *et al*, 2004). We studied another well-accepted aspect of ERα MISS, that is, ERα interaction with the tyrosine kinase src using the Duolink technique (Soderberg *et al*, 2006). We found that E_2_ (10^−8^ M) favored this interaction, whereas a 100-fold higher dose (E_4_ 10^−6^ M) was less efficient in inducing this aspect of MISS (Fig 7A). Importantly, when administrated together, the combination totally abrogated the ERα-src interaction, suggesting that, as shown above in endothelial cells, E_4_ was able to antagonize the action of E_2_ on ERα MISS. We also explored the impact of E_2_ 10^−8^ M, E_4_ 10^−6^ M, and their combination on the gene expression of MCF-7. As shown in Fig 7B, E_2_ 10^−8^ M and E_4_ 10^−6^ M similarly up-regulated the expression of genes containing ERE in their regulatory sequences, such as the gene regulated by estrogen in breast cancer 1 (GREB1) (Sun *et al*, 2007), the progesterone receptor (PR) (Kraus *et al*, 1993), and the chemokine (C-X-C motif) ligand 12 (CXCL12) (Boudot *et al*, 2011). Interestingly, and in striking contrast with the MISS effect, E_2_–E_4_ combination elicited the same induction than each isolated compound, showing no detectable interaction in these ERα nuclear actions.

## Discussion

Estetrol (E_4_), a physiological estrogen with four hydroxyl groups produced only by the fetal liver, appears to be human specific, but its physiological role is unknown. Furthermore, very few data are available concerning its molecular mechanisms of action. In this study, we demonstrate through *in vitro* and *in vivo* experiments that E_4_ is able to induce ERα transcriptional activity (about 100-fold above the doses of E_2_ required for the responses considered). Accordingly, the positioning of E_4_ in the ligand-binding pocket is very similar to that of E_2_, leading to a positioning of helix 12 and AF-2 availability that are nearly identical to that elicited by E_2_. Notably, although the affinity of E_4_ for ERα is 100-fold less than E_2_, the ERα complex with E_4_ is able to bind the important coactivator SRC3 as the complex with E_2_. We and others previously demonstrated that endometrial proliferation is highly dependent on the ERα nuclear actions, since this effect is abrogated in ERαAF-2^0^ and ERαAF-1^0^ mice (Abot *et al*, 2013), whereas it is fully preserved using a mouse with a point mutation of the palmitoylation site of ERα (C451A-ERα) that leads to membrane-specific loss of function of ERα (Adlanmerini *et al*, 2014). The potent atheroprotective effect observed in response to E_4_ also fits nicely not only with an ERα-dependent effect, as demonstrated by its abrogation in ERα^−/−^ mice, but also with the nuclear action of ERα. Indeed, we previously demonstrated that E_2_ failed to induce its atheroprotective action using AF-2^0^LDLR^−/−^ mice, highlighting the importance of nuclear/transcriptional actions of ERα for atheroprotection (Billon-Gales *et al*, 2011).

In contrast, E_4,_ even at high doses, is not able to elicit major endothelial actions known to be membrane ERα dependent, namely an increase in eNOS phosphorylation, in NO production, or an acceleration of reendothelialization (Chambliss *et al*, 2010; Adlanmerini *et al*, 2014). Furthermore, it antagonizes partially these MISS effects of ERα in response to E_2_. We also found that although E_4_ promotes some level of ERα-src interaction, E_2_/E_4_ combination does not promote any interaction. Already, H. Coelingh Bennink *et al* reported in the cancer-induced rat model that mammary tumor formation induced by DMBA treatment was stimulated by E_2_ and EE, but prevented by E_4_ (Coelingh Bennink *et al*, 2008a). Very recently, it has been demonstrated that E_2_ through a MISS effect enhanced the migration and invasiveness of human T47D breast carcinoma cells (Giretti *et al*, 2014). In contrast, E_4_ failed to stimulate and even antagonized the stimulation of T47D cells migration and invasion through matrigel by E_2_. According to our current understanding of MISS effects in breast cancer (Acconcia & Marino, 2011; Le Romancer *et al*, 2011), these data suggest that in this context E_4_ could have a safer profile than classic estrogens. Altogether, E_4_ appears to behave as a full or partial membrane ERα antagonist.

The structure as well as the conformation of ERα at the plasma membrane remains unclear, although palmitoylation appears to play an important role in its membrane localization and extranuclear-initiated actions (Acconcia *et al*, 2004; Adlanmerini *et al*, 2014). It thus appeared to us that comparing the physical interaction characteristics of these two estrogens, E_2_ and E_4_, in artificial membranes could shed some light to the lack of MISS action of E_4_. E_4_ was found to be almost as soluble as E_2_ in artificial membranes, ruling out the possibility that the lack of membrane signaling by E_4_ could be the result of its lack of availability in this cell compartment. In addition, whereas E_2_ was found to be in equilibrium between two orientations in the bilayer, E_4_ had a preferential orientation with its phenol group oriented toward interface and the three hydroxyl groups thus being at the hydrophobic core of the membrane. This orientation is rather counterintuitive, although an efficient intramolecular network of hydrogen bonds among the three D-ring OH groups might be masking their polarity more effectively than the lone 17β-OH in E_2_. The relationship between membrane orientation of an estrogen and its access to the ligand-binding site in membrane ERα, however, is at this point a matter of speculation, but it is clear that both E_2_ and E_4_ bind to ERα regardless of whether it is localized in the nucleus or the plasma membrane.

It is important to underline that the molecular mechanisms that mediate MISS effects of estrogen are far to be fully understood. The downstream target regulated by the ERα MISS involved various post-transcriptional modifications which probably highly differ between cell types. In endothelial cells, PI3K, Akt kinase, ERK1/2, striatin, and phosphorylation of eNOS have been described to be required for ERα MISS, whereas in vascular smooth muscle cells, expression and activity of several phosphatases such as MKP-1, SHP-1, PTEN, and PP2A mediate this pathway (Ueda & Karas, 2013). Since E_4_ is specific for humans and is produced only by the fetal liver, it is tempting to speculate that E_4_ might be conferring a very specific but important modulating effect of E_2_ action on fetal development, especially on brain development, as the nervous system appears to be largely influenced by MISS actions (Vasudevan & Pfaff, 2007).

Defect of E_4_ action via the membrane ERα pathway could also play a role on gene expression profiles and phenotypic effects of ERα action in organs that are dependent on both nuclear and membrane effects. Several authors proposed that nuclear action of ERα and of other transcription factors are regulated by MISS actions of estrogens (O'Malley & McGuire, 1968; Bjornstrom & Sjoberg, 2002; Lannigan, 2003; La Rosa *et al*, 2012), and the respective level of dependency of tissues on both nuclear and membrane effects could also be determined thanks to C451A-ERα and ERαAF-2^0^ mice. Although this cross talk was not observed for cell proliferation in uterus (Adlanmerini *et al*, 2014), it could be important in other tissues.

This original profile of ERα activation, uncoupling nuclear and membrane activation is, to the best of our knowledge, unique and characterizes E_4_ as a natural endogenous selective ER modulator (Table 4), reinforcing the idea that medical applications should be pursued further. Indeed, E_4_, in combination with a progestin, inhibits ovulation during the reproductive life (Coelingh Bennink *et al*, 2008c), or alleviates the climacteric symptoms after menopause (Holinka *et al*, 2008). As mentioned in the introduction, two recent phase 2 clinical trials evaluated the contraceptive efficacy of 5–20 mg E_4_ and levonorgestrel or drospirenone as a progestin. The first study evaluated ovulation inhibition in 91 women (18–35 year old) by measuring follicular size and endometrial thickness by ultrasound and evaluating the plasma levels of FSH, LH, E_2_, and progesterone. No ovulation was observed during the three cycles of treatment. The second study evaluated the bleeding profile in 330 young women over six cycles. An excellent bleeding and spotting profile clearly demonstrated the capacity of E_4_ to maintain a stable endometrium that was superior to the control group treated with E_2_ and dienogest. Lack of ovulation in all women was also verified by measuring the urinary excretion of pregnanediol, a progesterone metabolite. Remarkably, changes in SHBG, corticosteroid binding globulin (CBG), angiotensinogen, triglycerides, or coagulation proteins were minimal and considerably lower than in the comparator group receiving a combination of EE and drospirenone. Altogether, these experimental and clinical studies indicate that E_4_ should now be considered as a natural SERM. It is able to stimulate the endometrium, but it has no or only a minimal impact on the liver function. Dedicated experimental studies and randomized clinical trials of E_4_ are now needed, as better therapeutic alternatives are greatly needed by physicians and patients both in the field of oral contraception and as agents to replace the loss of beneficial estrogen effects resulting from the menopause.

**Table 4:** Current understanding of the impact of E_2_ and E_4_ on nuclear versus membrane initiated steroid signaling (MISS) ERα-mediated effects

Estrogens	Cell or tissue effects			
	Uterus	MCF-7		Endothelial cells
	Transcription/ proliferation	Transcription ERE dependent	Src-ERα interaction	Cell migration/ eNOS activation
E_2_	+++	+++	+++	+++
E_4_	+++	+++	+	0
E_2_ + E_4_	+++	+++	0	0/+
Prominent mechanism of action	Nuclear		Miss	

## Materials and Methods

### Expression purification and crystallization of ERα ligand-binding domain

ERα-LDB was expressed with a N-terminal Histidine tag in *E. coli* (BL21 DE3) and induced with isopropyl-β-d-thiogalactopyranoside (IPTG) for 16 h at 18°C. Cell pellets were lysed in 5 pellet volumes of lysis buffer [50 mM Tris pH7.6, 500 mM NaCl, 10% glycerol, 0.05% β-octyl glucoside, 10 mM imidazole, 5 mM β-mercaptoethanol, protease inhibitor (Roche) and 0.1 mg/ml lysozyme]. The lysates were centrifuged at 30,000 *g* for 30 min, and the supernatant was collected and loaded on a Ni-affinity resin. ERα-LDB protein was eluted with lysate buffer containing 500 mM imidazole. ERα-LDB was further purified on a size exclusion column. ERα was crystallized in complex with E_2_, E_3_ or E_4_, and GRIP peptide using a commercial screen formulation Index (Hampton Research) (Hsieh *et al*, 2006) Data collection was performed on single crystals at sector 19 (Structural Biology Center Collaborative Access Team at Agronome National Laboratory).

### Cell culture and transfection assays

MCF-7 cells were maintained in DMEM (Sigma-Aldrich) supplemented with 10% fetal calf serum (FCS) (Biowest) and antibiotics (Sigma-Aldrich) at 37°C in 5% CO_2_. One day before treatment, cells growing in 10 cm diameter dishes were placed in phenol red-free DMEM (Sigma-Aldrich) containing 2.5% charcoal-stripped FCS (Biowest). Cells were then treated for 24 h with E_2_ (10^−8^ M), E_4_ (10^−6^ M), combined treatment or ethanol.

HepG2 and HeLa cells were maintained in DMEM (Sigma-Aldrich) supplemented with 10% fetal calf serum (FCS) (Biowest) and antibiotics (Sigma-Aldrich) at 37°C in 5% CO_2_. Transfections were carried out using jetPEI reagent according to manufacturer's instructions (Polyplus). One day before transfection, cells were plated in 24-well plates at 50% confluence. One hour prior to transfection, the medium was replaced with phenol red-free DMEM (Sigma-Aldrich) containing 2.5% charcoal-stripped FCS (Biowest). Transfection was carried out with 100 ng of ERE-TK promoter driven renilla luciferase (luc) reporter, 100 ng of CMV-β galactosidase (Gal) internal control, and 50 ng of pCR3.1, pCR-ERα, pCR-ERα Δ79, or pCR-ERαAF-1^0^ expression vectors. Following an overnight incubation, cells were treated for 24 h with E_2_, E_4_, or ethanol (vehicle control). Cells were then harvested, and luciferase and β-galactosidase assays were performed as previously described (Penot *et al*, 2005).

### Mice

All procedures involving experimental animals were performed in accordance with the principles and guidelines established by the National Institute of Medical Research (INSERM) and were approved by the local Animal Care and Use Committee. ERα-null mice (ERα^−/−^) were generated as previously described (Billon-Gales *et al*, 2009) and were kindly provided by Pr P. Chambon (Strasbourg, France). To generate the double-deficient mice, LDLr^−/−^ female mice, purchased from Charles River (L'Arbresle, France), were crossed with ERα^+/−^ mice. The mice were anesthetized by injection of ketamine (100 mg/kg) and xylazine (10 mg/kg) by intraperitoneal route. To analyze E_4_ uterine action, C57Bl/6J were ovariectomized at 4 weeks of age and were subcutaneously injected with vehicle (castor oil), E_2_, or E_4_ at different doses 3 weeks later. Mice were sacrificed 6 or 24 h after a single estrogen injection and uteri were collected.

### Analysis of mRNA levels by RT-qPCR

Tissues were homogenized using a Precellys tissue homogenizer (Bertin Technol., Cedex, France), and total RNA from tissues was prepared using TRIzol (Invitrogen, Carlsbad, CA). One microgram of RNA was reverse transcribed (RT) at 25°C for 10 min and then at 37°C for 2 h in 20 μl final volume using the High Capacity cDNA reverse transcriptase kit (Applied Biosystems). For gene expression in uterus, the 96.96 Dynamic Arrays for the microfluidic BioMark system (Fluidigm Corporation, CA, USA) were used to study by high throughput qPCR the gene expression profile in 6.5 ng cDNA from each sample, as described previously (Abot *et al*, 2013). For gene expression in aorta, qPCR was performed using SsoFast EvaGreen Supermix (Bio-Rad) with primers validated by testing the PCR efficiency (Fontaine *et al*, 2013). Gene expression was quantified using the comparative C_t_ (threshold cycle) method.

Total RNA from MCF-7 cells was also extracted using TRIzol™ (Invitrogen) according to the manufacturer's instructions. cDNAs were generated using MMLV Reverse transcriptase (Invitrogen) and random hexamers (Promega, Madison, WI, USA). Quantitative RT-PCR was performed using the iQ SybrGreen supermix (BioRad, Hercules, CA, USA) on a BioRad MyiQ apparatus. Sequences of the primers used for cDNA amplification in the quantitative RT-PCR experiments are available upon request. Results were normalized to GAPDH expression.

### Uterus immunohistochemistry

Four-micrometer paraffin-embedded transverse sections from formalin fixed uterine specimens were dewaxed in toluene and rehydrated through acetone bath to deionized water. Antigen retrieval was performed in 10 mM citrate buffer pH 6.0 for 30 min in a water bath at 95°C. Cooled sections were then incubated in peroxidase blocking solution (Dako) to quench endogenous peroxidase activity. To block non-specific binding, sections were incubated in normal goat serum (Dako) for 20 min at room temperature. Primary antibodies were all rabbit polyclonal antibodies: anti-Ki-67 antigen (Thermo-scientific). Sections were incubated 50 min at room temperature with primary antibodies. The secondary antibody, biotinylated goat anti-rabbit immunoglobulins (Thermo-Scientific), was applied for 25 min at room temperature followed by an HRP-streptavidin solution (Dako) for 25 min. Peroxidase activity was revealed by 3,3′-diaminobenzidine tetrahydrochloride substrate (Dako). Finally, sections were counterstained with Harris hematoxylin, dehydrated and coverslipped. The luminal epithelial height (LEH) and stromal height (SH) were measured from the basal membrane to the apical surface. The values are the mean of ten measurements in each transverse uterus section.

### Analyses of atherosclerosis lesions

Bilateral ovariectomy was performed at 4 weeks of age. At 6 weeks of age, mice were switched to a hypercholesterolemic atherogenic diet (1.25% cholesterol, 6% fat, no cholate, TD96335, Harlan Teklad, Wisconsin) mixed with E_4_ (calculated to correspond to either 0.6 or 6 mg/kg/day) during 12 weeks. Over-night fasted mice were anesthetized, and blood was collected from the retro-orbital venous plexus. Lipid deposition size was evaluated at the aortic sinus as previously described (Billon-Gales *et al*, 2009). Briefly, each heart was frozen on a cryostat mount with OCT compound. One hundred 10-μm thick sections were prepared from the top of the left ventricle, where the aortic valves were first visible, up to a position in the aorta where the valve cusps were just disappearing from the field. After drying for 2 h, the sections were stained with oil red O and counterstained with Mayer's hematoxylin. Ten sections out of the 100, each separated by 90 μm, were used for specific morphometric evaluation of intimal lesions using a computerized Biocom morphometry system. The first and most proximal section to the heart was taken 90 μm distal to the point where the aorta first becomes rounded. The mean lesion size (expressed in μm^2^) in these 10 sections was used to evaluate the lesion size of each animal.

### Determination of plasma lipids

Total cholesterol was assayed using the CHOD-PAD kit (Horiba ABX, Montpellier, France). The high density lipoprotein (HDL) fraction was isolated from 10 μl of serum and assayed using the ‘C-HDL + Third generation’ kit (Roche, Lyon, France).

### Mouse carotid injury and quantification of reendothelialization

Bilateral ovariectomy was performed at 4 weeks of age, and concomitantly the mice received pellets implanted subcutaneously releasing either placebo, E_2_ (17β-estradiol 0.1 mg, 60 days release, i.e., 80 μg/kg/day, Innovative Research of America, Sarasota, FL) or an osmotic minipump releasing E_4_ (1 or 6 mg/kg/day). After 2 weeks treatment, carotid electric injury was performed as previously described (Brouchet *et al*, 2001) and reendothelialization was evaluated after 3 days. Briefly, surgery was carried out with a stereomicroscope (Nikon SMZ800), and the left common carotid artery was exposed via an anterior incision in the neck. The electric injury was applied to the distal part (3 mm precisely) of the common carotid artery with a bipolar microregulator. Three day postinjury, carotid arteries were stained with Evans blue dye and mounted with Kaiser's Glycerol gelatin (Merck). Images were acquired using DMR 300 Leica microscope using LAS V3.8 and ImageJ software. Percentage of reendothelialization was calculated relative to the initial deendothelialized area (Brouchet *et al*, 2001; Chambliss *et al*, 2010).

### Western blotting

Total proteins from aortae were separated on a 10% SDS/PAGE gel and transferred to a nitrocellulose membrane. The primary antibodies used are as follows: pSer1177-eNOS (612392; BD Bioscience), eNOS (610297; BD Bioscience), and β-actin (A2066; Sigma). Revelation was performed using an HRP-conjugated secondary antibody and visualized by ECL detection according to the manufacturer's instructions (Amersham Biosciences/GE Healthcare), using ChemiDoc Imaging System (Bio-Rad). Bands were quantified using ImageJ densitometry.

### Real-time NO production

Aorta from intact mice (10–12 weeks) was quickly harvested and maintained in 200 μl Krebs–Ringer oxygenated solution containing 2.5 mmol/l glucose at 37°C. A NO-specific amperometric probe [ISO-NOPF100; World Precision Instruments (WPI), Sarasota, FL] was implanted directly in the tissue, and NO release was monitored. The aorta was exposed to E_2_ (10^−8^ M) or E_4_ (10^−6 ^M) during 5 min. For cotreatment experiment, E_4_ (10^−6^ M) or vehicle (DMSO) was pre-incubated during 10 min prior to E_2_ (10^−8^ M) treatment. The concentration of NO gas in the tissue was measured in real time with the data acquisition system LabTrax (WPI) connected to the free radical analyzer Apollo1000 (WPI). Data acquisition and analysis were performed with DataTrax2 software (WPI). The NO-specific amperometric probe was calibrated as previously described (Knauf *et al*, 2001).

### Proximity Ligation Assay

The Proximity Ligation Assay (PLA) technology was developed by Olink Bioscience (Sweden) (Soderberg *et al*, 2006) and is commercialized by Sigma-Aldrich. For PLA, MCF-7 cells (5 × 10^4^ cells/ml) were grown on coverslips into 24-well plates in phenol red-free DMEM/F12 containing 5% charcoal-stripped FCS and were treated or not with E_2_ (10 nM) or E_4_ (1 μM) for 5 min. Cells were then fixed in 4% paraformaldehyde for 10 min and washed in large amount of PBS, and the coverslips were treated according to manufacturer's instructions (Duolink II Fluorescence, Olink Bioscience). Then, couple of primary antibodies rabbit anti-ERα (HC20 (Santa Cruz technology) and mouse anti-Src (B12, Santa Cruz Technology) was incubated overnight at 4°C in PBS with 0.2% triton and 0.5% non-fat milk. After washes, the PLA minus and plus probes (containing the secondary antibodies conjugated with complementary oligonucleotides) were added and incubated 1 h at 37°C. The next step allows the ligation of oligonucleotides if the two proteins are in close proximity thanks to the ligase during an incubation of 30 min at 37°C. After washes, the addition of nucleotides and polymerase allows amplification by rolling-circle amplification reaction using the ligated circle as a template during an incubation of 100 min at 37°C. The amplification solution also contains fluorescently labeled oligonucleotides that hybridize to the rolling-circle amplification product. The coverslips were let drying at room temperature in the dark and were mounted with Duolink II mounting Medium containing Dapi. The hybridized fluorescent slides were viewed under a Zeiss AxioImager Z1 microscope. Images were acquired under identical conditions at objective ×40. On each samples, at least 500 cells were counted. Analyses and quantifications of these samples were performed using ImageJ software that allows counting dots on 8 bits image and the plugin ‘Counter cells’ allows analyzing cells number.

**The paper explained**

**Problem**

Estetrol (E_4_) is an estrogen produced by the human fetal liver only during pregnancy. A recent clinical phase II study evaluating its contraceptive properties revealed that E_4_ did not change the levels of hepatic-derived proteins, including coagulation factors. Thus, at variance to classically used estrogens, it might not increase thrombo-embolic events. The molecular mechanism of action of E_4_ is essentially unknown, and the goal of this study was to define the nuclear/transcriptional actions versus the membrane/rapid actions in comparison to E_2_.

**Results**

In this study, we show that E_4_ is less potent than E_2_ to activate estrogen receptor alpha (ERα), but a high dose is able to modulate the transcriptional activity of ERα in the uterus, the proliferation of endometrial epithelium and to prevent atheroma. In contrast, E_4_ was not only devoid of effects on endothelial healing and eNOS activation, but it antagonized these E_2_ effects that are purely membrane ERα-dependent.

**Impact**

Thus, E_4_ appears not only as less potent estrogen than E_2_ but behaves as a natural selective ER modulator, and its spectrum of action as safe oral contraceptive or hormonal treatment of menopause should now be considered.

### Statistical analyses

Results are expressed as the mean ± SEM (Standard Error Mean). To test the effect of treatments, 1-way ANOVA was performed. To test the respective roles of treatment and genotype (ERα deficiency), a 2-way ANOVA was performed. When an interaction was observed between the two factors, the effect of treatment was studied in each genotype using a Bonferroni's *post hoc* test. A value of *P *< 0.05 was considered as statistically significant.
