# Supplementary material for: The uterine and vascular actions of estetrol delineate a distinctive profile of estrogen receptor α modulation, uncoupling nuclear and membrane activation
Source: EMBO Mol Med. 2014 Sep 11;6(10):1328–46. doi: 10.15252/emmm.201404112 (PMC4287935; doi:10.15252/emmm.201404112)
Supplement: Supplementary file 1 [file emmm0006-1328-sd1.pdf]

Supplementary Information

Material and Methods	2
Figure S1	3
Figure S2	4
Figure S3	5
Figure S4	6
	7
Table S1	8
Table S2	9

Determination of SRC3 binding affinity for ERα complexed with E_2, E_3 and E_4 (coactivator titration assay determining RCA). ERα LBD (1 nM) complexed with SA-Tb (fluorescence donor) was titrated against increasing concentration of Fl-SRC3 (fluorescence acceptor) in the presence of saturating concentration of various ligands (25 µM) and the ligand induced tr-FRET was measured as described previously (Jeyakumar et al. 2011). The diffusion enhanced-FRET corrected specific FRET values are shown. Data was analyzed by nonlinear regression with an equation for the sigmoidal dose response (variable slope) using GraphPad Prism 5, from which the EC50 values were determined. The RCA values were determined by the equation $EC_{50}^{[compound]}/EC_{50}^{E2}$ x 100. Three independent experiments were performed in replicate, and the results from one representative assay are shown.

Jeyakumar, M., K. E. Carlson, J. R. Gunther and J. A. Katzenellenbogen (2011). "Exploration of dimensions of estrogen potency: parsing ligand binding and coactivator binding affinities." J Biol Chem.

NMR experiments on model membrane systems:

To get insight in the orientation of the steroids, liposomes containing either E_4 or E_2 were prepared as previously described (Scheidt HA et al. 2010). They contained 1-palmitoyl-2-oleoyl-sn-glycero-3-phosphocholine (POPC) – sterol (E_2 or E_4) in a 8/2 molar ratio and were hydrated with D_2O at 50% w/w ratio. Liposomes containing either E_4 or E_2 were prepared as previously described (Scheidt HA et al. 2010). The liposomes (25 mg dry weight) was then placed in a 4 mm rotor and analyzed in a HR-MAS double resonance probe at 8 kHz spinning frequency and a temperature of 303 K on a Bruker Avance 500 MHz spectrometer. Using an original approach based on NOESY build up rates in liposomes, Scheidt *et al* previously reported that E_2 is stably inserted, although highly dynamic, into POPC model membranes (Scheidt et al. 2010). This method is based on the observation of proximities between the sterol resonances and the lipid resonances and on comparing E_2 phenolic proton proximities with POPC protons located at the bilayer interface or in the membrane center.). ^{1}H NOESY HRMAS buildup curves were obtained on E_2 and E_4 liposomes using mixing times of 1, 60, 150, 300 and 500 ms. The curves are linear with respect to the mixing time up to 150 ms, so that the NOE intensities at 150 ms may be taken as a good approximation of the cross relaxation rates. Intramolecular NOEs within the sterol contribute largely to the apparent intermolecular NOEs. Since the NOE build up curves are linear up to 150 ms, the NOESY cross peaks at this mixing time reflect the sterols behavior.
The solubility was assessed by measuring the intensities of the mobile sterol ^{1}H resonances (the insoluble sterol fraction did not show up in a ^{1}H-HRMAS spectrum but instead was visible in a ^{13}C CP MAS spectrum). Measurements were done either on low (50% w/w) or higher (500% w/w) hydration level for liposomes formed from initial molar steroid/POPC ratios of 2/8 and 1/9 and 0.5/ 9,5, respectively.

Scheidt HA, Badeau RM, Huster D 2010 Investigating the membrane orientation and transversal distribution of 17beta-estradiol in lipid membranes by solid-state NMR. Chemistry and physics of lipids 163:356-361

Relative Binding Affinity (RBA) Values binding to ERα and ERβ. The relative binding affinity of each estrogen for ERα and ERβ was determined by a competitive radiometric binding assay, as previously described (Carlson et al.1997). Each measurement represents mean ± SD of three separate experiments performed in replicate. Ki values were determined by the equation: (100/RBA) x Kd, where the Kd for E_2 is 0.2 nM for ERα and 0.5 nM for ERβ.

Carlson, K. E., I. Choi, A. Gee, B. S. Katzenellenbogen and J. A. Katzenellenbogen (1997). "Altered ligand binding properties and enhanced stability of a constitutively active estrogen receptor: evidence that an open pocket conformation is required for ligand interaction." Biochemistry **36**(48): 14897-14905.

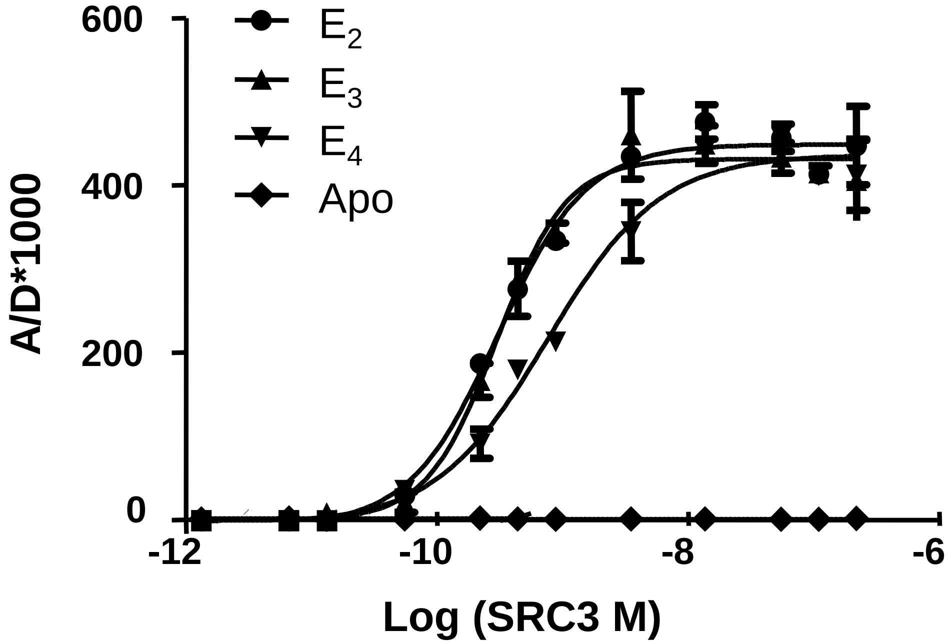

Supplementary Figure S1

Determination of SRC3 binding affinity for ERα complexed with E_2, E_3 and E_4 using coactivator titration assay determining RCA.

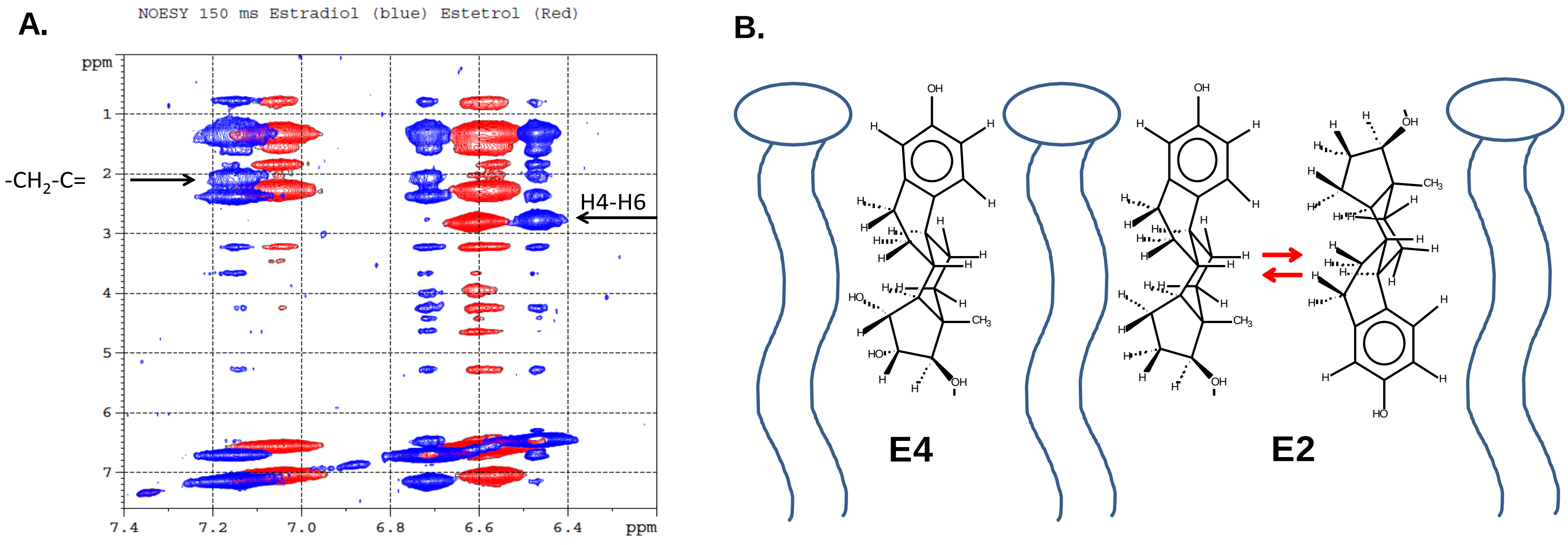

Supplementary Figure S2

A) 2D NOESY at 150 ms mixing time for E_2 (in blue) and E_4 (in red) . A superposition of the NOE contacts between the lipid resonances and E_2 and E_4 aromatic protons was observed. The arrow at 2.02ppm (left) shows the contacts between the sterol's aromatic protons H1, H2 and H4 and the lipid resonance at 2.02 ppm which corresponds to CH_2 protons adjacent to the POPC double bond, i.e. in the middle of the lipid chains (this chemical shift shows no overlap with other any sterol resonance and thus no contamination by intramolecular NOEs). It can clearly be seen that this NOE is stronger for E_2 than for E_4. This is confirmed by other intermolecular contacts.

B) Schematic drawing illustrating the membrane insertion of E_2 (in equilibrium between two orientations) and E_4 (one major orientation placing its phenolic ring at the bilayer interface) based on these NOE contacts. For both sterols the phenolic ring is majorily at the bilayer interface. However it is more dynamics for E_2 showing a fast equilibrium between two orientations, one of them placing the phenolic ring towards the bilayer center. E_4 on the contrary is more firmly anchored with its phenolic ring at the bilayer – water interface.

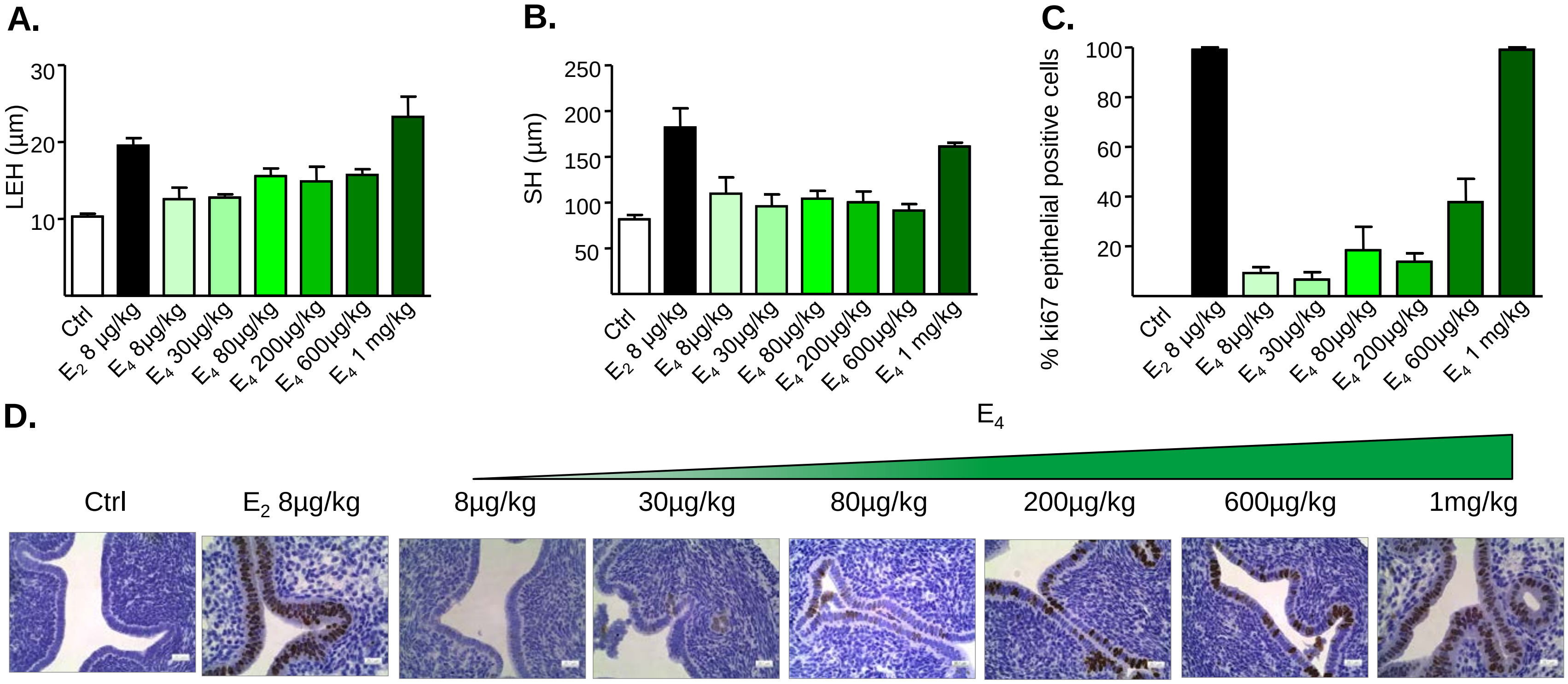

E.

P-values		E_2 8µg/kg	E_4 8µg/kg	E_4 30µg/kg	E_4 80µg/kg	E_4 200µg/kg	E_4 600µg/kg	E_4 1mg/kg
**LEH**	*vs* Ctrl	< 0.0001	ns	ns	ns	ns	0.0397	< 0.0001
	vs E2	-	0.0078	0.0107	ns	ns	ns	ns
**SH**	*vs* Ctrl	0.0003	ns	ns	ns	ns	ns	ns
	vs E2	-	0.0346	0.0062	0.0174	0.0043	0.0003	ns
**% Ki67**	*vs* Ctrl	< 0.0001	ns	ns	ns	ns	0.0087	< 0.0001
	vs E2	-	< 0.0001	< 0.0001	< 0.0001	< 0.0001	0.0006	ns

Supplementary Figure S3

Seven-week-old ovariectomized C57Bl/6J mice were subcutaneously injected with placebo (Ctrl, castor oil), 17β-estradiol (E_2, 8µg/kg) or estetrol (E_4, 8, 30, 80, 200, 600 µg/kg or 1 mg/kg) and were euthanized 24 hours after treatment. **(A-B)** luminal epithelial height (LEH) and stromal height (SH) were measured. **(C-D)** Ki-67 detection in transverse uterus sections (scale bar = 50µm) and percentage of Ki-67 positive epithelial cells were represented. Results are expressed as mean +/- SEM. To test the respective roles of each treatment, a 1-way ANOVA was performed and a Bonferroni's multiple comparison test **(E)** P-values of statistical test are indicated (n = 4 to 6 mice/group).

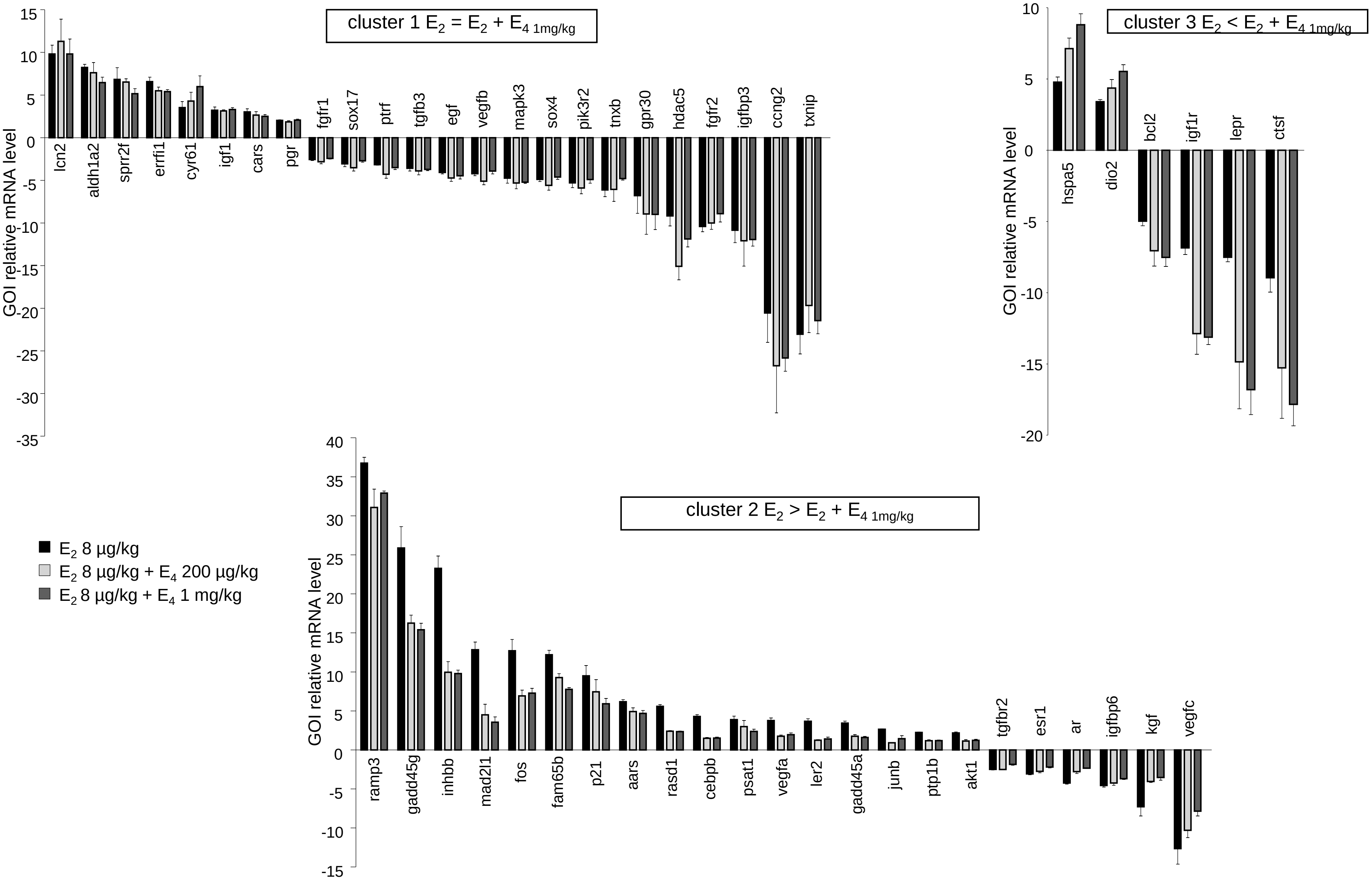

Supplementary Figure S4

Seven-week-old ovariectomized C57Bl/6J mice were subcutaneously injected with placebo (Ctrl, castor oil), 17β-estradiol (E_2, 8µg/kg) and/or estetrol (E_4, 200µg/kg or 1 mg/kg) and were euthanized 6 hours after treatment. mRNA levels of a set of genes from uterus that were regulated at least 2-fold by E_2 administration relative to placebo were measured by quantitative PCR and normalized to HPRT1 expression (n = 3 to 4 mice/group).

	RBA		Ki (nM)	
	ERα	ERβ	ERα	ERβ
E_2	[100]	[100]	0.2	0.5
E_3	6.8 ± 0.7	11.6 ± 0.6	2.94 ± 0.30	4.31 ± 0.22
E_4	0.65 ± 0.05	1.11 ± 0.16	30.8 ± 2.4	45.0 ± 6.5

Supplementary Table S1: Relative Binding Affinity (RBA) Values for E_2,E_3 and E_4 Binding to ERα and ERβ.

Name	ERα	
	EC_{50} (nM)	RCA
E_2	0.74 ± 0.06	[100]
E_3	0.75 ± 0.09	98.8 ± 3.5
E_4	1.7 ± 0.01	42.5 ± 3.6

Supplementary Table S2: EC_{50} and Relative Coactivator Binding Affinity (RCAs) of SRC3 for ERα Complexes with E_2, E_3 and E_4
