# Supplementary material for: The uterine and vascular actions of estetrol delineate a distinctive profile of estrogen receptor α modulation, uncoupling nuclear and membrane activation
Source: EMBO Mol Med. 2014 Sep 11;6(10):1328–46. doi: 10.15252/emmm.201404112 (PMC4287935; doi:10.15252/emmm.201404112)
Supplement: Supplementary file 2 [file emmm0006-1328-sd2.pdf]

EMBO Molecular Medicine

The uterine and vascular actions of Estetrol delineate a distinctive profile of Estrogen Receptor α modulation, uncoupling nuclear and membrane activation.

Anne Abot, Coralie Fontaine, Mélissa Buscato, Romain Solinhac, Gilles Flouriot, Aurélie Fabre, Anne Drougard, , Shyamalan Rajan, Muriel Laine, Alain Milon, Isabelle Muller, Daniel Henrion, Marine Adlanmerini, Marie-Cécile Valéra, Anne Gompel, Cécile Gerard, Christel Péqueux, Mélanie Mestdagt, Isabelle Raymond-Letron, Claude Knauf, François Ferriere, Philippe Valet, Pierre Gourdy, Benita S. Katzenellenbogen, John A. Katzenellenbogen, Françoise Lenfant, Geoffrey L. Greene, Jean-Michel Foidart and Jean-François Arnal

Corresponding author: Jean-François Arnal, Inserm U1048

Review timeline:

Submission date:	02 April 2014
Editorial Decision:	06 May 2014
Revision received:	18 July 2014
Editorial Decision:	26 July 2014
Revision received:	06 August 2014
Accepted:	08 August 2014

Transaction Report:

(Note: With the exception of the correction of typographical or spelling errors that could be a source of ambiguity, letters and reports are not edited. The original formatting of letters and referee reports may not be reflected in this compilation.)

Editor: Roberto Buccione

1st Editorial Decision 06 May 2014

Thank you for the submission of your manuscript to EMBO Molecular Medicine. We have now received comments from the three Reviewers whom we asked to evaluate your manuscript

You will see that all three Reviewers are quite supportive of you work, although they do raise a few issues that prevent us from considering publication at this time. I will not dwell into much detail, as the evaluations are self-explanatory. I would like, however, to mention the main points.

Reviewer 1 would like to see experiments showing the effects of E4 on phosphorylation of NOS via ER.

Reviewer 2 suggests that further experimentation is required to analyse the impact of E4 on endogenous ER and suggests an avenue to pursue this.

Reviewer 3 notes that in some instances the data do not fully support the conclusions. S/he further mentions that the clinical relevance of the findings should be further developed. Reviewer 3 also lists a number of other issues for your action.

I am especially concerned about the clinical relevance of your findings. One of the main reasons we decided to send your manuscript out for peer-review was that E4 emerges as a "SERM". Indeed, the accompanying manuscripts suggest its potential in the contraception arena. It is of the utmost

© EMBO

importance for the scope of our title that you provide data to better bridge your findings to the medical aspects, perhaps through your collaboration with the groups working on the trials. Without this significant improvement I am afraid I would agree with Reviewer 2, who notes that the manuscript appears too specialistic in its current form.

In conclusion, while publication of the paper cannot be considered at this stage, we would be prepared to consider a suitably revised submission, provided that the Reviewers' concerns and our requirements are fully addressed with further experimentation where required.

Please note that it is EMBO Molecular Medicine policy to allow a single round of revision only and that, therefore, acceptance or rejection of the manuscript will depend on the completeness of your responses included in the next, final version of the manuscript.

As you know, EMBO Molecular Medicine has a "scooping protection" policy, whereby similar findings that are published by others during review or revision are not a criterion for rejection. Nevertheless, I do ask you to get in touch with us after three months if you have not completed your revision, to update us on the status. Please also contact us as soon as possible if similar work is published elsewhere.

I look forward to receiving your revised manuscript.

***** Reviewer's comments *****

Referee #1 (Remarks):

Anne Abot et al. in this provocative, novel, and revealing study have explored the actions of estetrol (E4), a doubly hydroxylated derivative of estradiol (E2) that is made exclusively by the human fetus. They show with great care and clarity that E4 acts as a weakly potent but fully effective estrogen on the nuclear actions of estrogen receptors, ERalpha and ERbeta. It is as effective as E2 both on activated and repressed target genes in the uterus. Like E2, E4 allows for activation of both transcriptional activation functions AF-1 and AF-2. Like E2, E4 recruits coactivators. Most impressively E4 binding confers a 3-D atomic structure nearly identical to that conferred by E2 as determined by x-ray crystallography. In contrast to these effects of E4 on the nuclear actions of ERS, E4 is unable to activate the non-nuclear, membrane associated actions of ER on the vascular endothelium including endothial NO release and acceleration of endothial healing. Indeed, E4 appears to act as an antagonist of E2 actions on the membrane receptor.

The finding that E4 is an antagonist of E2 actions at a membrane site are unexpected, novel, and of substantial importance for the further understanding of estrogen action not only in the vascular system but also, for example, in the brain.

This review has only two minor suggestions concerning publication of this important work:

1) Why did not the authors also show that E4 was unable to stimulate and blocked E2 ability to stimulate phosphorylation of NO synthetase,? They had previously shown that mutant ER alpha unable to function at the membrane was unable to stimulate such phosphorylation. The absence of this experiment is strange given the completeness of the authors study of E4 action in the nucleus.

2) The discussion section is too long and can easily be shortened.

3) Please include the roles of BSK, JAK, and GLG

Referee #2 (Remarks):

This manuscript explores the functional role of E4 and the comparison with E2 in modulating ER effects in various contexts. The authors provide the crystal structure of ER LBD in the presence of E2 or E4 and show that although it has lower affinity for ER, it still produces similar co-factor binding docking regions. The impact of E4 on ER transcriptional activity is assessed in different cell

© EMBO

line models. Importantly E4 regulation on uterine genes is assessed and compared to E2 and finally, the role of E4 in atheroprotection and endothelial biology is assessed using nice systems.

Overall this is a good manuscript on an unexplored area of interest. The experiments are robust and well conducted. The findings are likely to be of interest to the community, albeit a reasonably specialized subset of the community.

- Why is the impact of E4 on endogenous ER not assessed in the cell line models, to definitely confirm signalling through ER-mediated gene transcription? Can the authors conduct an experiment where they hormone deprive cells that express endogenous ER (i.e. MCF-7 cells) and treat with E2 or E4 to assess ER function, potentially ER-DNA interactions (by ChIP) or expression of known ER target genes.

Referee #3 (Comments on Novelty/Model System):

The model system is adequate for the mechanistic questions addressed, however, clearer justification for use of atherosclerotic mice need to be provided in introduction, given the species differences in response to cardioprotective mechanisms of estradiol and metabolites.

Referee #3 (Remarks):

The manuscript of Abot et al details experiments to determine the molecular and cellular functionality of E4 as it compares to E2. The experiments undertaken by this group are methodologically sound, and do provide a good basis for understanding the mechanism of action of E4 in the uterus and vascular system. However, I do have several concerns and comments about the introduction, results and discussion. In particular there appears to be a tendency to overemphasize subtle results to confirm the Author's hypothesis.

Introduction:
Lines 25-28: While you state correctly that there are species differences with respect to E2 effects on the vascular system, and that mice are not a good pre-clinical model for humans, you do report data using mice pre-disposed to atherosclerosis (LDLr-/-). Please provide a good justification for using these mice to investigate a partial cardioprotective function of E2 here, instead of primate tissues or human cell lines such as HUVEC.

Results
Lines 77-78: A 2-fold difference in SRC3 binding is still statistically significant.
Lines 80-81: While ERalpha-E3 is almost as good as ERalpha-E2, while only 47% of ERalpha-E4 forms a complex with SRC3. Please revise the statement to reflect the actual data.

Lines 128-129: Most of the upregulated genes needed 100-fold higher E4; however 7 of 23 were activated at lower levels of E4, and again this is a gross oversimplification of Table E4. These are very important data with respect to the relative safety of E4 vs E2, and it is glossed over and hidden in supplemental tables. While the present figures are fine, these data should also be expressed as a tree dendigram of gene function by pathway analysis to demonstrate differential effects on gene pathways by E4 vs E2.
Line 161: Since E4 may be used as a COC, should also report effects of E4 and either Drospirenone or Levonorgestrel to increase clinical significance of these findings.

Line 205: From the data presented in Figure 6C, this demonstrates a partial inhibition or blunting of the E2-NO response. This is actually expected given the binding affinity data presented here. At most, E4 is a weak antagonist or partial antagonist of E2 MISS activity.

Discussion: Please modify to better reflect some of the more subtle effects of E4, i.e., line 254, E4 did not completely antagonize the effects of E2, and therefore is at most a partial antagonist.

Materials and Methods:
Line 375: It appears you performed qPCR with SyberGreen technology. Please confirm.

© EMBO

1st Revision - authors' response 18 July 2014

Response to reviewer 1.

We are grateful to the reviewer for his/her careful reading of our manuscript and the thoughtful comments.

1/ Why did not the authors also show that E4 was unable to stimulate and blocked E2 ability to stimulate phosphorylation of NO synthetase. They had previously shown that mutant ER alpha unable to function at the membrane was unable to stimulate such phosphorylation. The absence of this experiment is strange given the completeness of the authors study of E4 action in the nucleus.

1/ As requested by the reviewer, we now report the respective effects of E2 and E4, as well as their combination, on eNOS phosphorylation in isolated aortae. We fully agree with the reviewer that this new approach reinforces the concept which demonstrates absence of MISS of ERα by E4 in endothelial cells and complement the data on NO production in mouse aorta. These data are presented as new Figure 6B.

2/ The discussion section is too long and can easily be shortened.

Discussion has been shortened and has been rewritten in order to highlight the clinical relevance of our findings.

3/ Please include the roles of BSK, JAK, and GLG

The role of each author is now described, as well as that of new authors who participated to the new data.

Response to reviewer 2.

We are grateful to the reviewer for his/her careful reading of our manuscript and the thoughtful comments.

Why is the impact of E4 on endogenous ER not assessed in the cell line models, to definitely confirm signalling through ER-mediated gene transcription? Can the authors conduct an experiment where they hormone deprive cells that express endogenous ER (i.e. MCF-7 cells) and treat with E2 or E4 to assess ER function, potentially ER-DNA interactions (by ChIP) or expression of known ER target genes.

As suggested by the reviewer, new experiments have been conducted in MCF-7 cells treated with either solvent, E4, E2, or their combination.

- We demonstrated that in this cell type, E4 is less efficient than E2 in mediating MISS of ERα since E4 promoted ERα-*src* interaction, a well recognized MISS ERα effect, less efficiently than does E2.
- We also examined the expression of genes known to be ERE-dependent in MCF-7, and we found that E4 induces their expression demonstrating that as in the uterus high dose of E4 is able to activate ERα nuclear action.
- Importantly, we report that E_4 antagonizes E_2 MISS-dependent effects (as previously described in the endothelium), whereas E_4 does not interfere with E2-induced ERα - dependent gene expression, with similar nuclear actions of E2, E4 or their combination.

Taken together, these results presented in a new Figure 7, allow us to conclude that in MCF-7 cells, E4 is not able to induce a full estrogenic response, but that its MISS effects are substantially blunted and that furthermore, E4 attenuates the MISS effect of E2. As it is widely accepted that extranuclear ERα MISS actions promote migration and/or proliferation of breast cancer cells (Acconcia & Marino, 2011, Le Romancer et al, 2011), this suggests a safer profile for E_4 concerning the most

© EMBO

dreaded side effect of estrogen use. This point is now discussed in the new version of the manuscript, along with several other studies that support this conclusion (Coelingh Bennink H.J.T., 2008) (Giretti et al, 2014).

Acconcia F, Marino M (2011) The Effects of 17beta estradiol in Cancer are Mediated by Estrogen Receptor Signaling at the Plasma Membrane. *Frontiers in physiology* 2:30

Le Romancer M, Poulard C, Cohen P, Sentis S, Renoir JM, Corbo L (2011) Cracking the estrogen receptor's posttranslational code in breast tumors. *Endocr Rev* 32: 597-622

Coelingh Bennink H.J.T. SC, Simoncini T., Genazzani A., Kubista E. (2008) Estetrol, a pregnancy specific human steroid, prevents and suppresses mammary tumor growth in a rat model. *Climacteric: the journal of the International Menopause Society* Suppl. 1:29

Giretti MS, Montt Guevara MM, Cecchi E, Mannella P, Palla G, Spina S, Bernacchi G, Di Bello S, Genazzani AR, Genazzani AD, Simoncini T (2014) Effects of Estetrol on Migration and Invasion in T47-D Breast Cancer Cells through the Actin Cytoskeleton. *Frontiers in endocrinology* 5:80

Response to reviewer 3.

We are grateful to the reviewer for his/her careful reading of our manuscript and the thoughtful comments.

Lines 25-28: While you state correctly that there are species differences with respect to E2 effects on the vascular system, and that mice are not a good pre-clinical model for humans, you do report data using mice pre-disposed to atherosclerosis (LDLr-/-). Please provide a good justification for using these mice to investigate a partial cardioprotective function of E2 here, instead of primate tissues or human cell lines such as HUVEC.

We reported that mice are not a good pre-clinical model in order to study hepatic factors since this parameter is species-dependent (Valera, 2012). However, mice are appropriate to investigate vascular effects and $LDLR^{-/-}$ mice are a widely accepted model of atheroma (Mallat & Tedgui, 2007; Weber et al, 2008) and the only one that gives access to the molecular mechanisms (here the role of ERα in the E4 action). This point is now highlighted in the new version of the manuscript (lines 175-176).

Mallat Z, Tedgu A (2007) Cytokines as regulators of atherosclerosis in murine models. *Current drug targets* 8: 1264-1272

Valera MC, Gratacap MP, Gourdy P, Lenfant F, Cabou C, Toutain CE, Marcellin M, Saint Laurent N, Sie P, Sixou M, Arnal JF, Payrastre B (2012) Chronic estradiol treatment reduces platelet responses and protects mice from thromboembolism through the hematopoietic estrogen receptor alpha. *Blood* 120: 1703-1712

Webe C, Zernecke A, Libby P (2008) The multifaceted contributions of leukocyte subsets to atherosclerosis: lessons from mouse models. *Nature reviews Immunology* 8: 802-815

Lines 77-78: A 2-fold difference in SRC3 binding is still statistically significant.
Lines 80-81: While ERalpha-E3 is almost as good as ERalpha-E2, while only 47% of ERalpha-E4 forms a complex with SRC3. Please revise the statement to reflect the actual data.

According to the reviewer's remark, interpretation of the data on SRC3 interaction has been changed as follows: " *In this assay, E3-ERα and E2-ERα have essentially identical affinities for SRC3, and the affinity of E4-ERα, while half that of E2-ERα, is still in the low nanomolar range (Figure E1 and Table E2).*"

Lines 128-129: Most of the upregulated genes needed 100-fold higher E4; however 7 of 23 were activated at lower levels of E4, and again this is a gross oversimplification of Table E4. These are very important data with respect to the relative safety of E4 vs E2, and it is

© EMBO

glossed over and hidden in supplemental tables. While the present figures are fine, these data should also be expressed as a tree dendigram of gene function by pathway analysis to demonstrate differential effects on gene pathways by E4 vs E2.

We agree with the reviewer that the dose response of E4 on uterus gene expression have to be more highlighted. Expanded Table 3 and Table 4 appear now as new Table 1 and Table 2 in the new version of the manuscript. In addition, and according to the reviewer's comment, interpretation of the data has been modified as followed: "*In most cases, compared to E_2, E_4 required a 100-fold higher dose (i.e., 1 mg/kg) to optimally activate the transcription of target genes (Table 1), although 7 of the 23 studied genes were activated at lower levels of E4. Concerning down regulated genes, a dose of 80 µg/kg of E_4 was sufficient to induce the maximal action (Table 2)*.

As the strategy used in order to evalute the transcriptional response to E4 in uterus was to analyse the quantitative changes of a selected set of genes known to be regulated by E2 (and not a global approach), we believe that tree dendigram of gene function by pathway analysis would not provide a suitable representation.

Line 161: Since E4 may be used as a COC, should also report effects of E4 and either Drospirenone or Levonorgestrel to increase clinical significance of these findings.

This question is definitively an important one, and the combination of E4 with drospirenone or levonorgestrel has been directly evaluated in 2 clinical trials. The main conclusions are now reported in the new discussion section of the manuscript.

5/ Line 205: From the data presented in Figure 6C, this demonstrates a partial inhibition or blunting of the E2-NO response. This is actually expected given the binding affinity data presented here. At most, E4 is a weak antagonist or partial antagonist of E2 MISS activity.
Discussion: Please modify to better reflect some of the more subtle effects of E4, i.e., line 254, E4 did not completely antagonize the effects of E2, and therefore is at most a partial antagonist.

We agree with the reviewer that E4 did not completely antagonize the effects of E2, and therefore is at most a partial antagonist. This point has been corrected (line 215 and line 268) in the new version of the manuscript. Importantly, this is true for the MISS affects, but not for the nuclear actions, and this is now not only demonstrated in the uterine/nuclear actions on the one hand, and on the endothelial/MISS actions on the other hand, but it is also demonstrated in the MCF7 cells (new Figure 7) where both aspects (MISS and nuclear) can be analyzed in the same cellular model.

Materials and Methods:
Line 375: It appears you performed qPCR with SyberGreen technology. Please confirm.

We performed qPCR using SsoFast EvaGreen Supermix (BioRad, Hercules, CA, USA) technology on tissue (aorta and uterus) experiments and using the iQ SybrGreen supermix (BioRad, Hercules, CA, USA) on MCF-7 cells experiment: this point is now specified in the material and method section of the manuscript.

© EMBO

2nd Editorial Decision 26 July 2014

Thank you for the submission of your revised manuscript to EMBO Molecular Medicine. We have now received the enclosed reports from the Reviewers that were asked to re-assess it. As you will see the Reviewers are now globally supportive and I am pleased to inform you that we will be able to accept your manuscript pending the following final amendments:

1) As per our Author Guidelines, the description of all reported data that includes statistical testing must state the name of the statistical test used to generate error bars and P values, the number (n) of independent experiments underlying each data point (not replicate measures of one sample), and the actual P value for each test (not merely 'significant' or 'P < 0.05').

2) Every published paper now includes a 'Synopsis' to further enhance discoverability. Synopses are displayed on the journal webpage and are freely accessible to all readers. They include a short standfirst - to be written by the editor - as well as 2-5 one-sentence bullet points that summarise the paper (to be written by the author). Please provide the short list of bullet points that summarise the key NEW findings. The bullet points should be designed to be complementary to the abstract - i.e. not repeat the same text. We encourage inclusion of key acronyms and quantitative information. Please use the passive voice. Please attach these in a separate file or send them by email, we will incorporate them accordingly.

3) It is essential to include high-quality files for all figures, which will be used for the production process. I note that the quality of the images in Fig. 5A could be improved (i.e. blocky/blurry); this is apparent, for example, when zooming in. Also, the blot images in figure 6B appear of rather low quality and too contrasted. Please provide better figures as these issues could lead to problems when the production team tries to resize these images for the final manuscript.

4) We are now encouraging the publication of source data, particularly for electrophoretic gels and blots, with the aim of making primary data more accessible and transparent to the reader. Would you be willing to provide a PDF file per figure that contains the original, uncropped and unprocessed scans of all or at least the key gels used in the manuscript? The PDF files should be labelled with the appropriate figure/panel number, and should have molecular weight markers; further annotation may be useful but is not essential. The PDF files will be published online with the article as supplementary "Source Data" files. If you have any questions regarding this just contact me.

Please submit your revised manuscript within two weeks. I look forward to seeing a revised form of your manuscript as soon as possible.

I look forward to reading a new revised version of your manuscript as soon as possible.

***** Reviewer's comments *****

Referee #1 (Remarks):

This revised ms is now very well documented. It adds an important chapter to the understanding of estrogen receptor action at the membrane and presents the first example of a ligand for the membrane receptor that is at least a partial antagonist.

Referee #2 (Remarks):

The authors have addressed my concerns.

2nd Revision - authors' response 06 August 2014

We thank you as well as the reviewers for the positive response concerning of our manuscript entitled “The uterine and vascular actions of Estetrol delineate a distinctive profile of Estrogen

© EMBO

Receptor α modulation, uncoupling nuclear and membrane activation" by Anne Abot *et al.* Please find enclosed a revised form of our manuscript in which we have included the following modification as requested:

1) We have now included the name of the statistical test, the number of mice and/or independent experiments and the actual *P* value for each data that includes statistical test.

2) Please find enclosed as an attached file named "synopsis" the bullet points that summarize the paper.

3) Figure 5A and Figure 6B have been modified using better quality files.

4) We have now provided a file containing the original scans of the gels used to perform the quantification in Figure 6B.

We hope that you will find the manuscript now suitable for publication in EMBO molecular medicine. We are looking forward hearing from you.

© EMBO
