# Supplementary material for: The uterine and vascular actions of estetrol delineate a distinctive profile of estrogen receptor α modulation, uncoupling nuclear and membrane activation
Source: EMBO Mol Med. 2014 Sep 11;6(10):1328–46. doi: 10.15252/emmm.201404112 (PMC4287935; doi:10.15252/emmm.201404112)
Supplement: Supplementary file 3 [file emmm0006-1328-sd3.pdf]

Experiment 1 (n = 3)

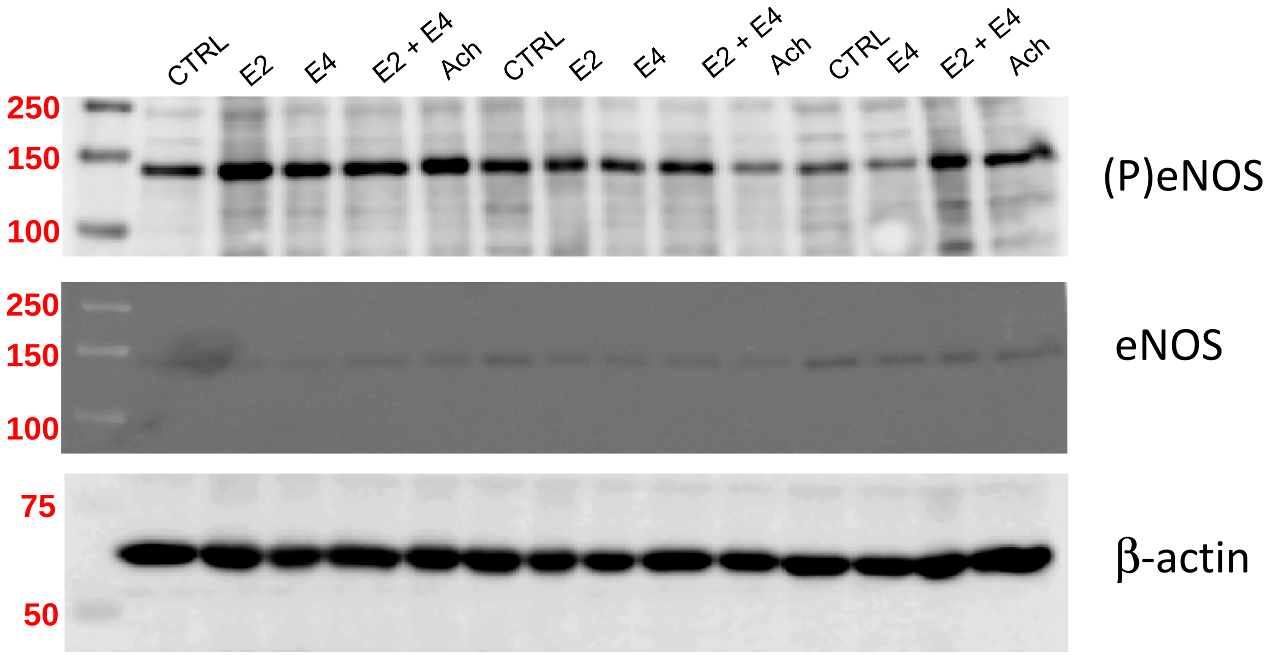

Experiment 2 (n = 3)

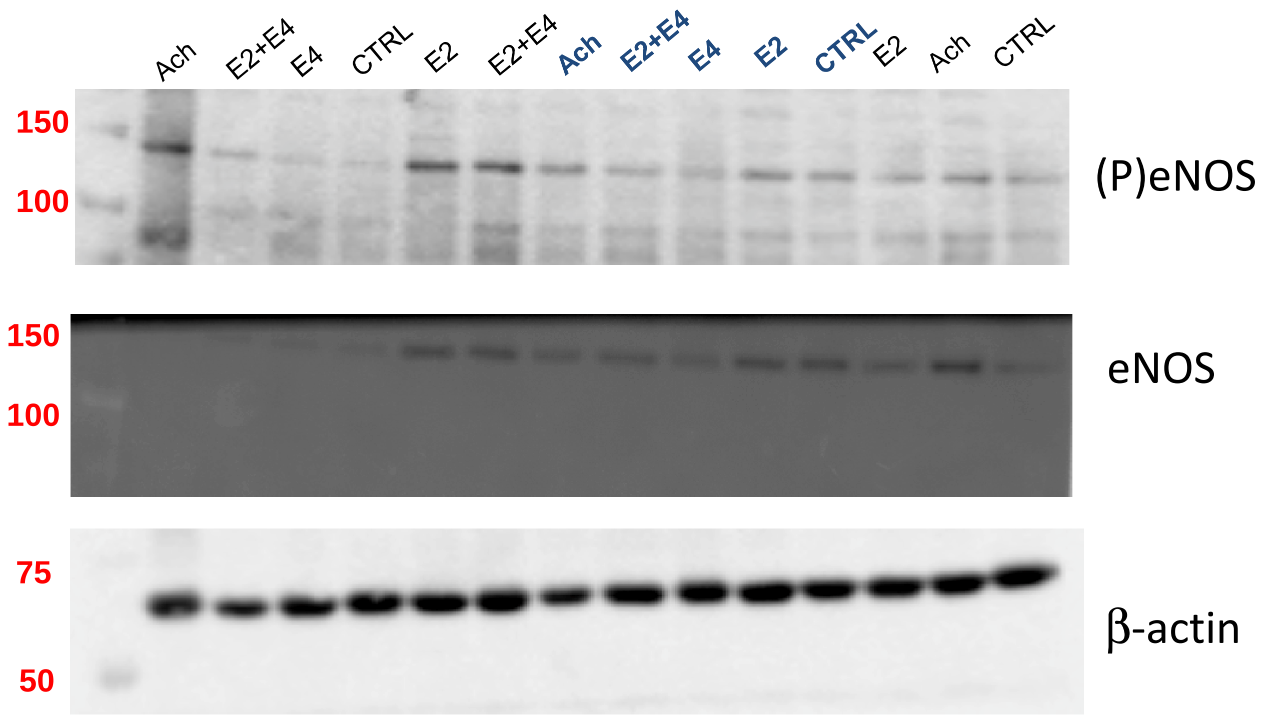

Abot *et al.*
Data source Figure6B

Experiment 3 (n = 2)

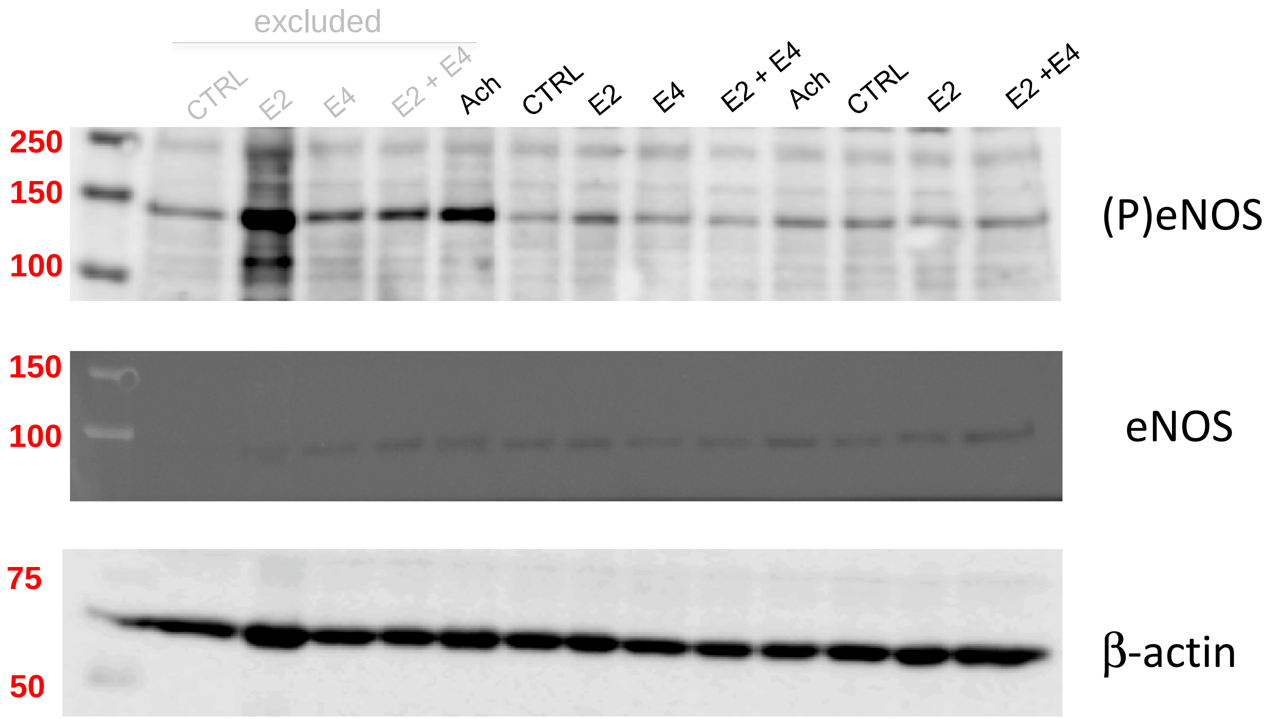

Abot *et al*.
Data source Figure6B
